# Behavioural and computational methods reveal differential effects for how delayed and rapid onset antidepressants effect decision making in rats

**DOI:** 10.1016/j.euroneuro.2017.09.008

**Published:** 2017-12

**Authors:** Claire A. Hales, Conor J. Houghton, Emma S.J. Robinson

**Affiliations:** a School of Physiology, Pharmacology & Neuroscience, Faculty of Biomedical Sciences, University of Bristol, Bristol BS8 1TD, UK; b Department of Computer Science, Faculty of Engineering, University of Bristol, Bristol BS8 1UB, UK

**Keywords:** Antidepressants, Behaviour, Diffusion model, Judgement bias, Ketamine, Rodent

## Abstract

Major depressive disorder (MDD) is one of the most prevalent psychiatric disorders. Until the recent discovery of the rapid onset antidepressant action of ketamine, pharmacological treatments for MDD were limited to conventional antidepressant drugs with delayed clinical efficacy. Using a judgement bias task, this study has investigated whether the temporal differences observed in patients would be reflected in affective biases and decision making behaviour in rodents. The diffusion model was also used to investigate the underlying decision making processes. Positive biases were induced in this task over timeframes that mirror the rapid versus delayed antidepressant efficacy of the drugs in clinical populations. Diffusion modelling revealed that the antidepressants tested also have different effects on decision making processes, suggesting they may act through different neurobiological substrates. This combination of behaviour and computational modelling may provide a useful approach to further investigate the mechanisms underlying rapid antidepressant effect and assess potential new treatments.

## 1 Introduction

In major depressive disorder (MDD), impairments in emotional processing lead to biases in cognitive processes (Murphy et al., 1999, Robinson et al., 2011, Roiser et al., 2012). The importance of these biases in MDD was first proposed by Beck in his cognitive theory of depression (Beck, 1976). More recently, affective biases have been linked to the development, maintenance and treatment of MDD (Teasdale, 1983, Teasdale, 1988, Beck, 2008, Harmer et al., 2009a, Kircanski et al., 2012). Processes including attention, memory, emotional interpretation and decision making have been shown to be negatively biased in people suffering from MDD (see Mathews and MacLeod, 2005; Clark et al., 2009; Gotlib and Joormann, 2010 for in-depth reviews). Treatment with antidepressants, including acute doses, induce positive biases in emotional processing in healthy controls (Harmer et al., 2003, Harmer et al., 2004, Harmer et al., 2011) and patients (Harmer et al., 2009b). However, this does not match the subjective experience of patients, where changes in mood are only reported several weeks after starting conventional antidepressant treatment (Anderson et al., 2000). In contrast, patients experience a subjective improvement in mood less than two hours after administration of the rapid-acting antidepressant ketamine (Zarate et al., 2006).

Understanding the processes which underlie delayed versus rapid onset antidepressant action is important if new faster acting treatments are to be developed. Different theories exist to explain the delayed onset of action of conventional antidepressants (Duman and Monteggia, 2006, Harmer et al., 2009a, Harmer, 2010, Duman and Li, 2012, Wang et al., 2015). The different time course of efficacy has been related to ketamine's ability to quickly increase synaptogenesis and density and function of spine synapses via rapid glutamatergic effects on intracellular signalling pathways (Duman et al., 2012). This is in comparison to the longer-term neuroplasticity and neurogenic changes that are produced by slow-onset adaptation of signalling pathways through conventional antidepressants acting on neuromodulatory (e.g. serotonergic) systems (Duman and Voleti, 2012). However, in rodent behavioural tests such as the forced swim test, both ketamine (Browne and Lucki, 2013) and conventional antidepressants (Porsolt et al., 1977, Cryan and Holmes, 2005) demonstrate efficacy after acute administration. Another commonly used rodent test for antidepressant efficacy, the sucrose preference test, can differentiate between delayed and rapid onset antidepressants but only when used in combination with a chronic stress manipulation (Willner, 2005, Browne and Lucki, 2013). This highlights the limitations of current animal models used to quantify depression-like behaviour, and concerns have been raised that the poor translation between pre-clinical studies and clinical benefits may relate to these issues (for discussion see Nestler et al., 2002; Cryan and Slattery, 2007; Nestler and Hyman, 2010; Berton et al., 2012; O'Leary and Cryan, 2013). The assessment of animal models of depression against the criteria of face, construct and predictive validity indicates that these assays may be limited to tests of monoaminergic antidepressant efficacy and fail to achieve either construct or predictive validity (Willner, 1984, Belzung and Lemoine, 2011). Identification of an animal test that could dissociate delayed and rapid antidepressant onset of action without the need for chronic stress procedures would provide a vital tool for the drug discovery process.

Building on research in humans, tests to study affective biases in animals have been developed, which include the affective bias test (Stuart et al., 2013, Stuart et al., 2015) and judgement bias tasks (see Hales et al., 2014 for a review). Evidence from studies using phenotypic models and drug treatments suggest that in non-human species these tasks can be used to quantify similar neuropsychological processes linked to affective biases. The judgement bias task is designed to more closely mirror the type of neuropsychological impairments seen in depression (Hales et al., 2014, Robinson and Roiser, 2016) and therefore achieves better face validity than traditional tests. The cause of depression remains unknown and therefore achieving construct validity is more difficult. Evidence from animals in putative depression-like states following chronic stress manipulations have revealed pessimistic behaviour in the judgement bias task (Harding et al., 2004, Papciak et al., 2013, Rygula et al., 2013) which is similar to the negative interpretation biases observed in depressed patients (e.g. Butler and Mathews, 1983; Nunn et al., 1997; Voncken et al., 2007). We have previously shown effects on biases in this task with chronic monoaminergic antidepressant treatment (Anderson et al., 2013), whilst experiments to date have generally failed to observe consistent effects with conventional antidepressants following acute administration (Anderson et al., 2013, Rygula et al., 2014a).

In the affective bias test in rats, a dissociation between delayed and rapid onset antidepressants has been shown in terms of their effects on learning and memory (Stuart et al., 2013, Stuart et al., 2015). Where conventional antidepressants can induce positive biases associated with new learning but lack the ability to attenuate previously acquired negative biases, ketamine was observed to have the opposite effect. This study focuses on investigating the predictive validity of the judgement bias task, specifically comparing the efficacy and time course of effects for delayed versus rapid onset antidepressants on judgement bias, and whether this concurs with their reported clinical outcomes in patients. We hypothesised that this task may provide a novel behavioural approach which could be used to dissociate between delayed and rapid onset antidepressants.

The diffusion model (Ratcliff, 1978, Ratcliff and Rouder, 1998) provides insight into the processes that underlie decision making by parameterising the subtle differences in shapes of response time (RT) distributions from behavioural two-choice decision making data. These parameters are associated with psychologically meaningful processes (Ratcliff, 1978). The diffusion model assumes that decision making begins at a relative starting point (*zr*) that is located somewhere between two boundaries (*a* and *0*), each representing one of the two alternative responses. Information accumulates over time from the starting point until one or other threshold is reached. Therefore, the starting point determines the amount of information that is required before a decision is made. The rate of this information accumulation (known as the drift rate, *v*) is governed by the quality of the stimuli, in that if incoming information is of high quality, the drift rate will be steeper and so a boundary (and hence decision) will be reached more quickly. Therefore, changes in the drift rate reflect changes in discrimination difficulty (when compared between trial types) or perceptual sensitivity (when compared across different conditions). Other parameters in the model include the time taken for non-decision processes (*t*_*0*_), parameters quantifying the variability in starting point, drift rate and non-decision time (*szr*, *sv* and *st*_*0*_ respectively) and a parameter that accounts for possible differences in the speed of response execution between the two choices (*d*).

Advantages of using a diffusion modelling approach over and above traditional analyses of behavioural data include: being able to disentangle the different factors that may alter decision making (e.g. response conservatism, perceptual factors or the influence of various biases; (Voss et al., 2013a, Voss et al., 2013b); using maximal information from behavioural data to provide a more thorough interpretation of results (as both RT and accuracy data are incorporated together in the model; White et al., 2009, White et al., 2010; Voss et al., 2013a); and increased sensitivity to detecting experimentally induced changes that may be split across both RT and accuracy in behavioural data (White et al., 2010).

To investigate whether a reward-based judgement bias task can dissociate between delayed and rapid onset antidepressants we tested the effects of acute treatments with conventional, delayed onset antidepressants: fluoxetine (a selective serotonin reuptake inhibitor; SSRI), reboxetine (a noradrenaline reuptake inhibitor, NRI) and venlafaxine (a serotonin and noradrenaline reuptake inhibitor, SNRI); versus the rapid-acting antidepressant, ketamine (an N-methyl-D-aspartate (NMDA) receptor antagonist) and phencyclidine (PCP), another NMDA receptor antagonist with no known antidepressant properties. For comparison, we also tested two psychostimulants, amphetamine and cocaine, which are known to induce hedonic effects following acute administration. Following the acute drug studies, a final experiment investigated the effect of chronic treatment (three weeks) with fluoxetine. We used a reward-based ambiguous cue interpretation task to measure judgement bias, and applied the diffusion model to behavioural results to add to the interpretation of the data (Hales et al., 2016).

## 2 Experimental procedures

### 2.1 Subjects and apparatus

Three cohorts of male Lister Hooded rats (cohort 1: *n*=16; cohort 2: *n*=18; cohort 3: *n*=16) were used (Harlan, UK). Rats weighed ~270 to 300 g at the start of training, and ~400 to 500 g at the start of experimental manipulations. They were pair-housed with environmental enrichment consisting of a red Perspex house, a cardboard tube and a cotton rope suspended across the cage lid. Rats were kept under temperature (19–23 °C) and humidity (45–65%) controlled conditions on a 12-h reverse lighting cycle (lights off at 08:00 h). Water was available *ad libitum* in the home cage, but rats were maintained at no less than 90% of their free-feeding body weight by restricting access to laboratory chow (LabDiet, PMI Nutrition International) to ~18 g per rat per day. All procedures were carried out under local institutional guidelines (University of Bristol Animal Welfare and Ethical Review Board) and in accordance with the UK Animals (Scientific Procedures) Act 1986. During experiments all efforts were made to minimise suffering, and at the end of experiments rats were euthanised by giving an overdose of sodium pentobarbitone. Behavioural testing was carried out between 0800 and 1800 h, using standard rat operant chambers (Med Associates, Sandown Scientific, UK). For details of operant chamber configuration, see Supplementary Materials and Methods.

### 2.2 Behavioural task

Animals were tested using a high versus low reward version of the ambiguous interpretation task as previously reported in Hales et al. (2016). Rats were trained to associate a correct response to two different auditory tones with either a high or low value reward. Responses made following a midpoint ambiguous tone were used to measure judgement bias.

#### 2.2.1 Training: cohort 1

Rats (*n*=16) in cohort 1 were trained to associate one tone (2 or 8 kHz at 75 and 64 dB respectively; counterbalanced across rats, designated high reward) with a large food reward (four 45 mg reward pellets; Test Diet, Sandown Scientific, UK) and the other tone (8 or 2 kHz, the opposite of the high reward tone, designated low reward) with a small food reward (one 45 mg reward pellet) if they pressed the associated lever (either left or right, counterbalanced across rats) during the 20 s tone. Table S1 contains a summary of training stages used. Once trained, these animals were used to test the acute effects of three conventional antidepressants – fluoxetine, reboxetine and venlafaxine.

#### 2.2.2 Training: cohorts 2 and 3

As rats in cohort 1 tended to show an innate bias towards the higher frequency tone and preference for the higher value reward (See Figure S6 for details), the second and third cohorts were not trained using a fully counter-balanced design. Instead, the 2 kHz tone was associated with the high reward for all rats (*n*=18/*n*=16). This reduced variability and improved sensitivity to detect altered responding to the midpoint ambiguous tone. Any innate biases were accounted for by using the animals own baseline to normalise data for cognitive bias index (CBI; see Statistical Analysis for details). All other training remained the same as for cohort 1 (See Table S1). Once trained, cohort 2 was used to test the acute effects of ketamine, PCP, amphetamine and cocaine. After the last acute study, rats were given at least two weeks washout before being split into two groups to allow for chronic treatment with fluoxetine (1.0 mg/kg daily) or vehicle. Cohort 3 were used for a replication study testing the effect of a single acute dose of 1.0 mg/kg ketamine.

#### 2.2.3 Ambiguous cue interpretation testing

Baseline sessions (100 trials: 50 high and 50 low reward tones; pseudorandomly) were conducted on Monday and Thursday. Probe test sessions (120 trials: 40 high reward, 40 low reward, and 40 ambiguous midpoint tones; pseudorandomly) were conducted on Tuesday and Friday. The ambiguous midpoint tone was made up of one of two tones (20×4999 Hz and 20×5001 Hz at 70 dB). The outcomes associated with the ambiguous tones were the same as the reference tone they were closer to, resulting in a random reinforcement of the midpoint tone. This meant a specific outcome should not be learnt for the midpoint tone, and ensured continued responding throughout the experiments. Responses to either of the two midpoint tones were analysed together. For further task details see Supplementary Materials and Methods. Rats were considered trained when they maintained stable responding for three consecutive days, and were excluded from analyses if they failed to maintain >60% accuracy during experiments.

### 2.3 Experimental design

#### 2.3.1 Acute drug treatments

Each acute dose-response study used a within-subject fully counterbalanced drug treatment schedule (See Table S2 for details of individual treatments). All drugs were dissolved in 0.9% sterile saline in a dose volume of 1 ml/kg and given by intraperitoneal injection using a low-stress, non-restrained technique (Stuart and Robinson, 2015). For testing the effect of acute antidepressant treatment, fluoxetine (0.3, 1.0 mg/kg), reboxetine (0.3, 1.0 mg/kg), venlafaxine (1.0, 3.0 mg/kg) or vehicle (0.0 mg/kg) were administered 30 min prior to the probe test session (all purchased from Tocris, UK). For all other studies, the experimenter was blinded to drug dose. For acute drug studies in cohort 2, each dose-response occurred across two weeks with three drug doses plus vehicle (0.0 mg/kg). Ketamine (0.3, 1.0, 3.0 mg/kg; *t*=−60 min), amphetamine (0.1, 0.3, 1.0 mg/kg; *t*=−15 min) and cocaine (0.3, 1.0, 3.0 mg/kg; *t*=−10 min) were purchased from Sigma-Aldrich, UK; PCP (0.3, 1.0, 3.0 mg/kg; *t*=−40 min) was purchased from Tocris, UK. In cohort 3, the ketamine replication (1.0 mg/kg, *t*=−60 min; Sigma-Aldrich, UK) occurred over one week. Drug doses were selected based on previous rodent behavioural studies (Stuart et al., 2013, Stuart et al., 2015, Benn and Robinson, 2014). Rats experienced at least one week re-baseline (five baseline sessions) between each experimental manipulation.

#### 2.3.2 Chronic fluoxetine treatment

A between-subjects study design was used, and the experiment was split into three parts: (1) a pre-drug week, (2) three weeks of drug treatment, and (3) one week post-drug testing. Rats were split into control (0.9% sterile saline vehicle) or fluoxetine (1.0 mg/kg) groups based on performance (matched for all analysed behavioural variables) during the pre-drug week. Rats were dosed daily at least 3 h following the end of behavioural testing by subcutaneous injection, commencing the Monday of first drug week and ending on the Thursday of final drug week.

### 2.4 Modelling

The diffusion model (Ratcliff, 1978) provides insight into the cognitive processes that underlie decision making on two-choice tasks by fitting parameters that correspond to different aspects of the decision making process (see Ratcliff and Rouder, 1998 for a detailed description of the model). This means it can distinguish between different factors that may affect decision making (e.g. interpretation of sensory information or the influence of biases), as these map on to different parameters. The model takes into account the full shape of response time (RT) distributions and accuracy data simultaneously, meaning maximal information can be extracted from behavioural data. Since the model describes RT distributions as well as accuracy data, behaviours with the same accuracy, for example, can correspond to different model parameters, reflecting different RT distributions. This means the diffusion model is more sensitive to detecting subtle changes, particularly if experimental manipulations cause changes that manifest across both RT and accuracy measures (Voss et al., 2004).

The diffusion model was fit using fast-dm-30 (Voss and Voss, 2007, Voss and Voss, 2008, Voss et al., 2010, Voss et al., 2015) to individual rats’ data from probe test sessions. Model fitting and validation followed that described previously (Hales et al., 2016), and further details are provided in Supplementary Methods and Materials.

### 2.5 Statistical analysis

CBI was used as a measure of judgement bias in response to the midpoint tone. CBI was calculated by subtracting the proportion of responses made on the low reward lever from the proportion of responses made on the high reward lever. This created a score between −1 and 1, where negative values represent a negative bias and positive values a positive bias. Change from baseline in CBI was then calculated for all experimental manipulations. For acute drug studies this was calculated as follows: vehicle (0.0mg/kg) probe test CBI−drug dose probe test CBI and for the chronic manipulation: average of the two pre-drug probe test CBI-drug/post drug probe test session CBI

This was calculated to take into account individual differences in baseline bias, and to make directional changes caused by drug treatments clearer. Although individuals within a cohort were variable regarding their CBI scores at baseline, performance was consistent across repeated sessions (compare the vehicle/pre-test percentage positive responses panels in Supplementary Figures S2–S5 – these show the behavioural data used to calculate CBI). To provide a value for vehicle probe test sessions for this measure, the population average for the vehicle (0.0 mg/kg) probe test was taken away from each individual rats’ CBI score for the same session. This allowed this measure to be analysed with repeated measures analysis of variance (ANOVA) with session as the within-subjects factor (and group as the between subjects factor for the chronic manipulation). Based on our *a priori* hypotheses, change from baseline in CBI was also analysed with one-sample *t*-tests with test values of zero (representing no change from the vehicle or control probe test session) for each drug dose in acute studies. For the chronic manipulation, change from baseline in CBI was also analysed in a separate analysis by week (to reduce the higher variability in this study due to lower *n* number per group) by taking the average of the two probe test sessions for that week.

Response latency and percentages of positive responses, omissions and premature responses were also analysed (see Table S3 for details of these). For acute manipulations, all measures were analysed with repeated measures analysis of variance (ANOVAs) with session and tone as the within-subjects factors. For the chronic manipulation, mixed ANOVAs were performed with two repeated measures (session and tone) as the within-subjects factor and group as the between-subjects factor. This was done initially after the pre-drug week for the first two probe sessions to check that groups were matched for all analysed behavioural variables, and then subsequent analysis following completion of the study was also conducted using all three experimental periods (pre-drug, drug and post-drug).

Diffusion model parameters analysed were change from baseline in relative starting point (*zr;* as described for change from baseline in CBI), drift rate (*v*) and boundary separation (*a*) for the midpoint tone. For the chronic manipulation, parameter values from individual sessions from each part (pre-drug, drug and post-drug) were averaged.

Paired *t*-tests or independent samples *t*-tests were performed as post-hoc tests if significant effects were established. Huynh-Feldt corrections were used to adjust for violations of the sphericity assumption, Levene's test was used to correct for inequality of variances, and Bonferroni correction was applied for multiple comparisons. All statistical tests were conducted using SPSS 21.0.0.0 for Windows (IBM SPSS Statistics) with *α*=0.05. Results are reported with the ANOVA *F*-value (degrees of freedom, error) and *p*-value as well as any post-hoc *p*-values. All graphs were made using Graphpad Prism 5.03 for Windows (Graphpad Software, USA).

## 3 Results

### 3.1 Acute effects of conventional, delayed onset antidepressants

**Figure 1:**
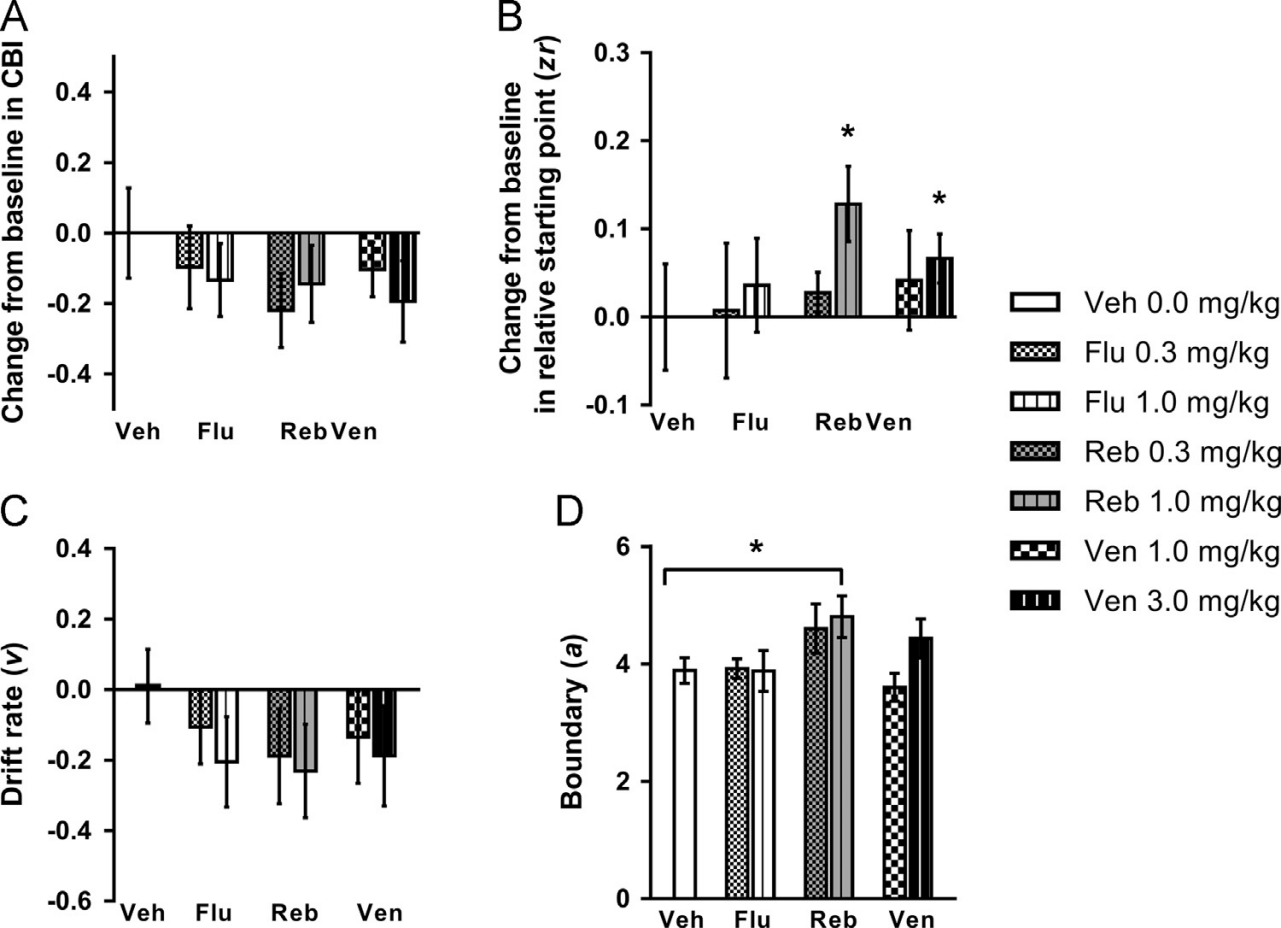
The effect of acute treatment with three antidepressants on behaviour and modelling of judgement bias. Fluoxetine (0.3, 1.0 mg/kg), reboxetine (0.3, 1.0 mg/kg), venlafaxine (1.0, 3.0 mg/kg) or saline vehicle (0.0 mg/kg) were administered acutely prior to testing on the judgement bias task. (A) None of the antidepressants tested at any dose induced a positive change in cognitive bias index (CBI) for the midpoint tone. Although not significant, reboxetine (0.3 mg/kg) showed a tendency to cause a negative change in CBI. (B/C/D) Behavioural data were modelled using the diffusion model and parameters corresponding to the relative starting point (*zr*), drift rate (*v*) and boundary separation (*a*) were analysed. (B) The higher doses of reboxetine (1.0 mg/kg) and venlafaxine (3.0 mg/kg) caused a positive change in *zr*. (C) There were no effects on *v*. (D) The higher dose of reboxetine (1.0 mg/kg) only caused a significant increase in *a*. Change from baseline (vehicle session) measures were calculated to take into account individual differences in underlying bias. Data shown are for the midpoint tone only, and represent mean ± SEM. *n*=9, 30 min pre-treatment. *p<0.05, ^#^*p*≤0.07. Veh – vehicle; Flu – fluoxetine; Reb – reboxetine; Ven – venlafaxine.

Nine rats attained criteria and were included in the analysis. None of the drugs tested significantly altered judgement bias (no main effect of session: *F*_3.175,25.396_=0.725, *p*=0.554; Figure 1A), although there was a tendency for 0.3 mg/kg reboxetine to cause a negative change from baseline in cognitive bias index (CBI; one sample *t*-test: *p*=0.070; Figure 1A). The 1.0 mg/kg dose of reboxetine increased response latency for the midpoint and low reward tones (Figure S1A). There were no other effects on behavioural measures (Figure S1B–D). Diffusion modelling revealed that the higher doses of reboxetine (1.0 mg/kg) and venlafaxine (3.0 mg/kg) caused a positive change from baseline in relative starting point (one sample *t*-tests: *p*s≤0.043; Figure 1B). There were no effects on drift rate (Figure 1C), but the boundary was increased compared to vehicle for 1.0 mg/kg reboxetine (main effect of session: *F*_6,42_=3.574, *p*=0.006 and pairwise comparison: *p*=0.032; Figure 1D). Reboxetine (1.0 mg/kg) caused a decrease in the variability of the decision starting point (Table S4).

### 3.2 Acute effects of NMDA receptor antagonists

**Figure 2:**
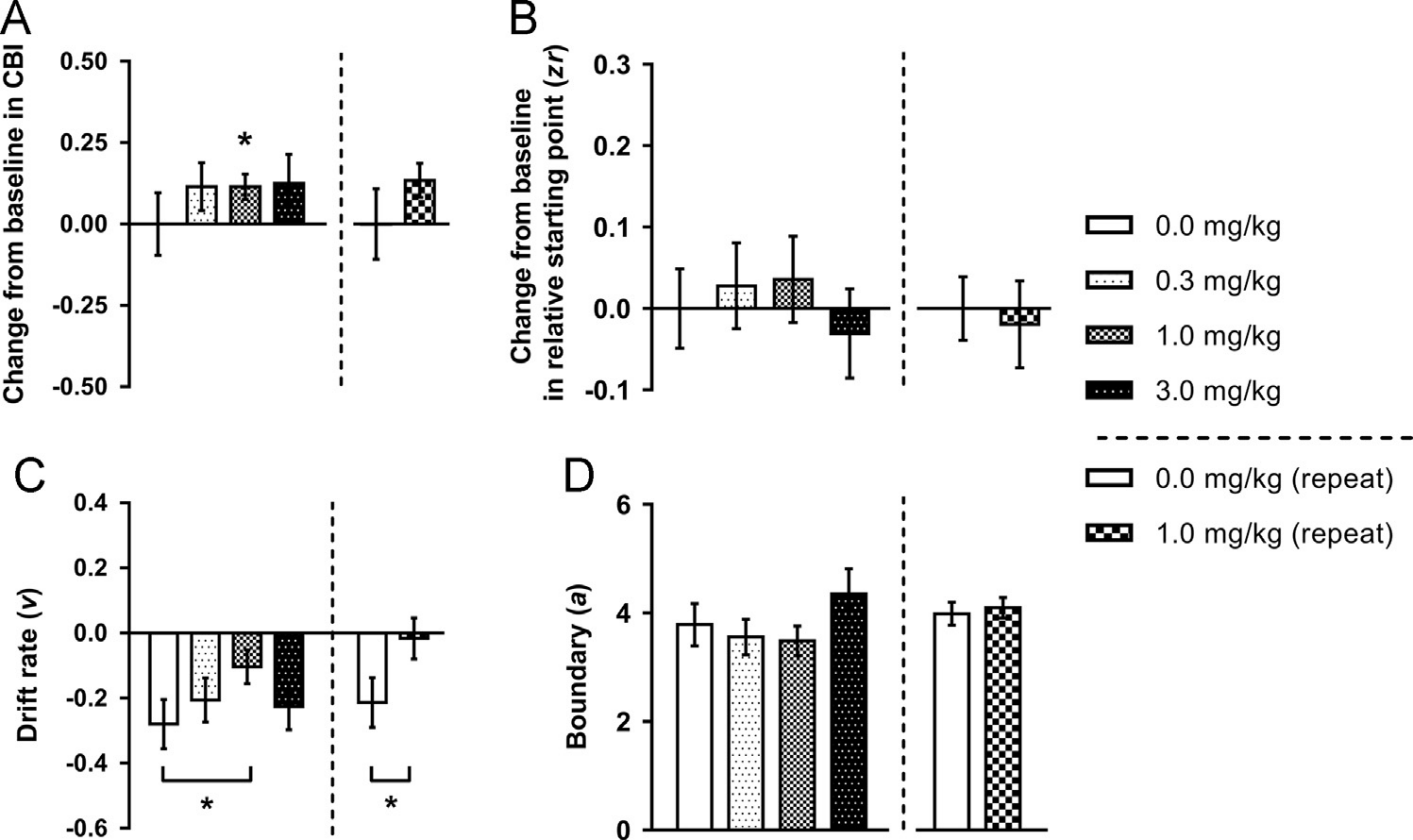
The effect of acute treatment with ketamine on behaviour and modelling of judgement bias. Ketamine (0.0, 0.3, 1.0, 3.0 mg/kg) was administered acutely prior to testing on the task. Behavioural data were modelled using the diffusion model and parameters corresponding to the relative starting point (*zr*), drift rate (*v*) and boundary separation (*a*) were analysed. (A) Acute treatment with ketamine (1.0 mg/kg), a rapid acting antidepressant, induced a positive bias at the midpoint tone. (B) Ketamine had no effect on *zr*. (C) The positive bias induced by 1.0 mg/kg ketamine is reflected by a more positive drift rate (*v*). (D) Acute treatment with ketamine did not alter (*a*). Change from baseline (0.0 mg/kg session) measures were calculated to take into account individual differences in underlying bias. Data shown are for the midpoint tone only, and represent mean±SEM. *n*=13, 60 min pre-treatment. *p<0.05.

Thirteen rats met criteria to be included in the analysis of the ketamine dose response. There was no overall main effect of ketamine on change from baseline in CBI (*F*_2.114,25.372_=0.953, *p*=0.403), but the 1.0 mg/kg dose showed a positive change from baseline in CBI measured using the one-sample *t*-test (*p*=0.012; Figure 2A). The other two doses caused changes of a similar magnitude but with higher variability (0.3 mg/kg: *p*=0.145; 3.0 mg/kg: *p*=0.187). There were no other effects on behavioural measures (Figure S2). Diffusion modelling revealed the drift rate was less negative compared to vehicle for the 1.0 mg/kg dose (main effect of session: *F*_3,36_=2.918, *p*=0.047 and post-hoc test: *p*=0.034; Figure 2C). Ketamine did not alter any other diffusion model parameters (Figure 2B/D; Table S4).

Fifteen rats were included in the single dose ketamine replication study. There was a session*tone interaction for percentage of positive responses (*F*_2,28_=3.367, *p*=0.049) which post-hoc tests revealed to be because ketamine (1.0 mg/kg) caused an increase in responses made on the high reward lever for the midpoint tone only (*p*=0.022; Figure S2). This reduction in negative judgement bias was also apparent in the change from baseline in CBI (one-sample *t*-test: *p*=0.022; Figure 2A). There were no changes to other behavioural measures (Figure S2). Diffusion model results were also replicated, where 1.0 mg/kg ketamine caused the drift rate to become less negative (main effect of session: *F*_1,14_=4.926, *p*=0.043 and post-hoc test: p=0.047; Figure 2C), but did not alter other parameters (Figure 2B/D; Table S4).

**Figure 3:**
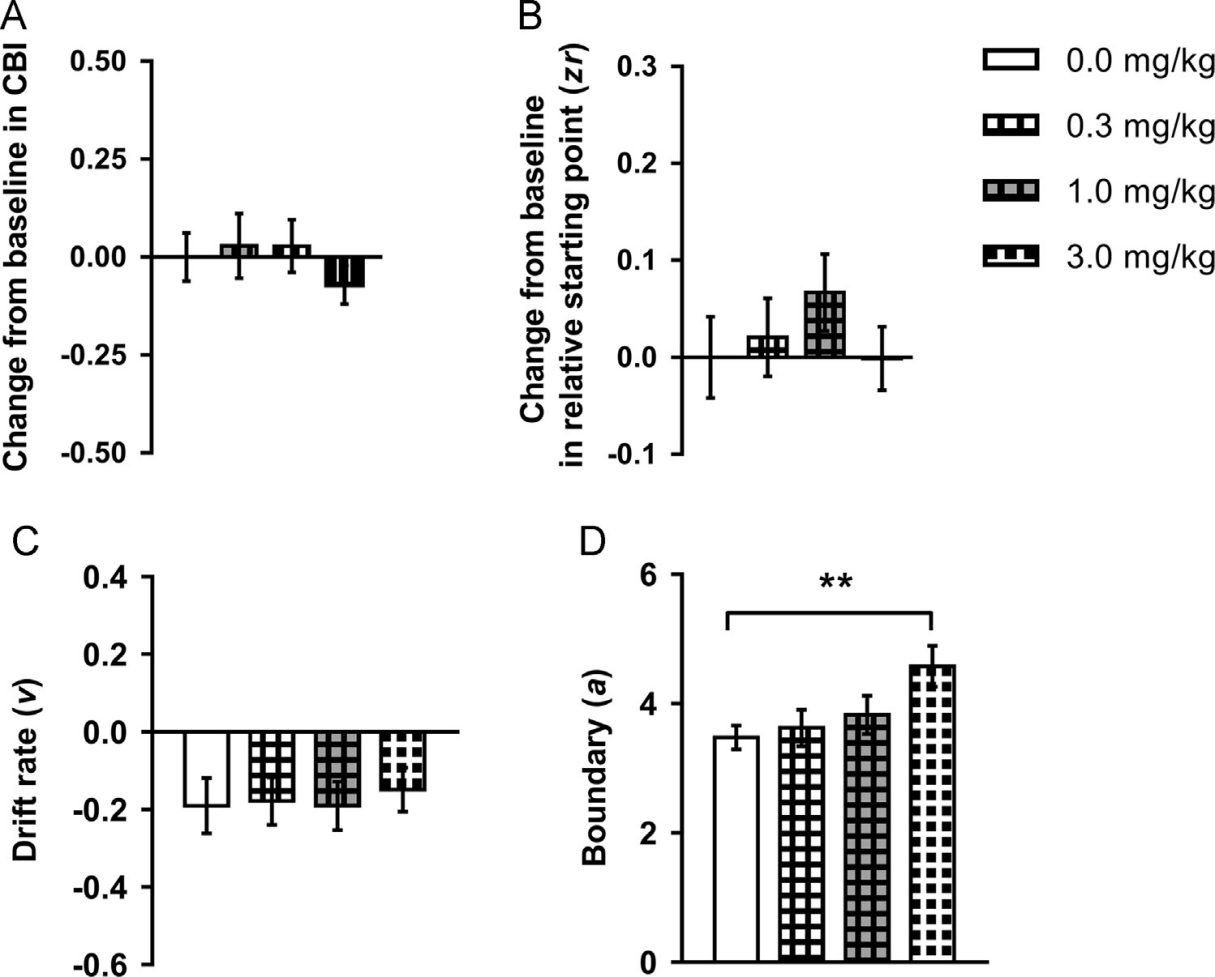
The effect of acute treatment with phencyclidine on behaviour and modelling of judgement bias. Phencyclidine (PCP; 0.0, 0.3, 1.0, 3.0 mg/kg) was administered acutely prior to testing on the task. Behavioural data were modelled using the diffusion model and parameters corresponding to the relative starting point (*zr*), drift rate (*v*) and boundary separation (*a*) were analysed. (A) Acute treatment with PCP had no effect on judgement bias. (B/C) PCP did not alter *zr* or *v*. (D) The highest dose of PCP (3.0 mg/kg) caused a significant increase in *a*. Change from baseline (0.0 mg/kg session) measures were calculated to take into account individual differences in underlying bias. Data shown are for the midpoint tone only, and represent mean±SEM. *n*=16, 40 min pre-treatment. **p<0.01.

Sixteen rats were included in the analysis for the PCP dose response study. No effects on CBI were observed for any of the doses tested (main effect of session: *F*_3,45_=0.493, *p*=0.689; one-sample *t*-tests: *p*s≥0.170; Figure 3A). The highest dose (3.0 mg/kg) caused slower response latencies for all tones (Figure S3A) and increased percentage omissions for the midpoint and low reward tones (Figure S3C), while 1.0 mg/kg PCP increased premature responding (Figure S3D). Consistent with the increased response latency, the highest dose of PCP (3.0 mg/kg) increased boundary separation (main effect of session: *F*_3,45_=5.775, *p*=0.002 and post-hoc test: *p*=0.002; Figure 3D) and the non-decision reaction time (Table S4). There were no other effects on diffusion model parameters (Figure 3B/C and Table S4).

### 3.3 Effect of chronic treatment with fluoxetine

**Figure 4:**
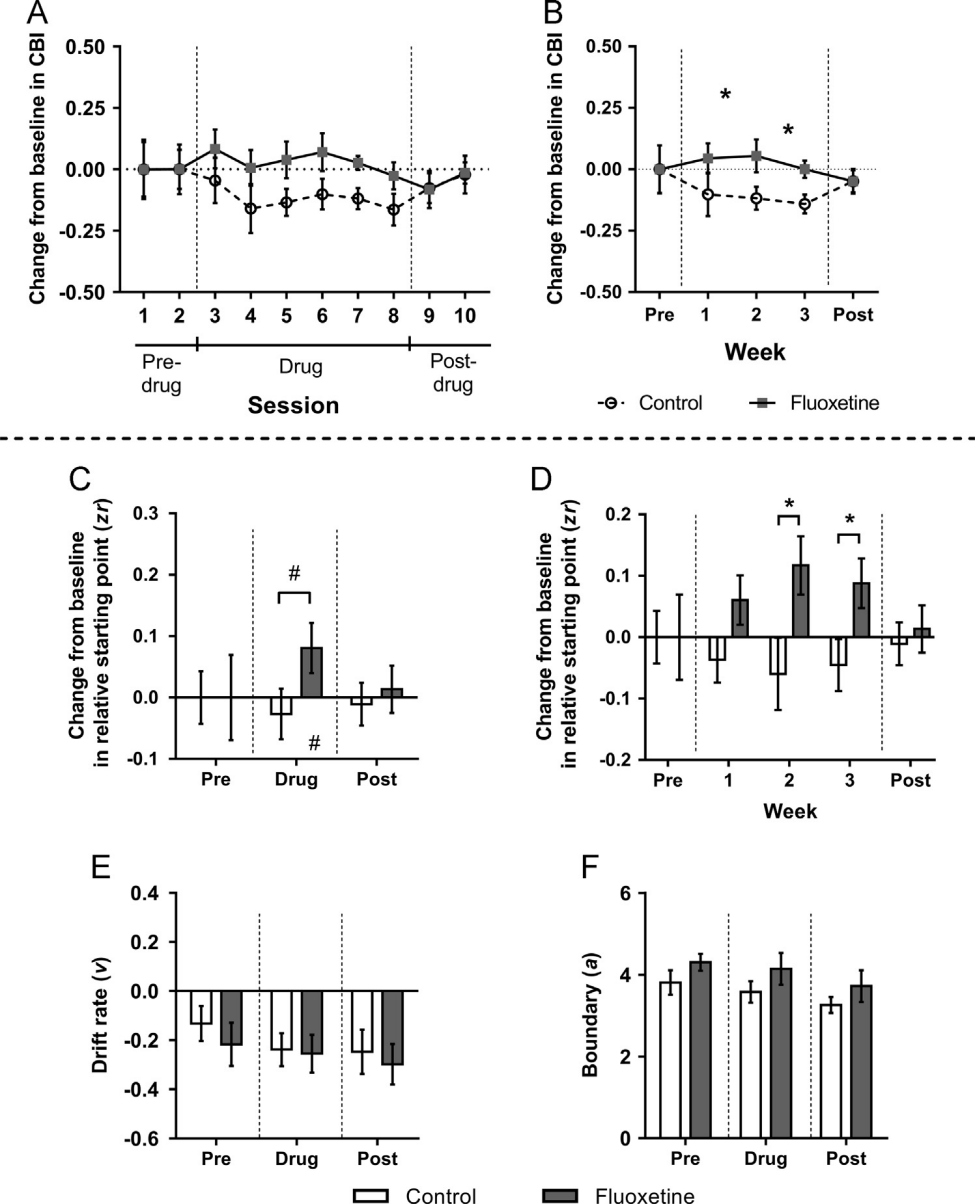
The effect of chronic treatment with an antidepressant on behaviour and modelling of judgement bias. Rats assigned to the chronic antidepressant group experienced subcutaneous injections of fluoxetine (1.0 mg/kg) daily for three weeks, whilst control rats experienced daily subcutaneous injections of saline vehicle (0.0 mg/kg). Twice weekly test sessions were conducted one week prior to treatment (pre; sessions 1–2), for the three weeks during treatment (drug; sessions 3–8) and for one following the end of treatment (post; sessions 9–10). There were no significant differences between groups during the pre-drug period for any measure. (A) Rats in the fluoxetine-treated group had a significantly more positive change from baseline in cognitive bias index (CBI) compared to controls during the drug period only. (B) Analysing each week of treatment shows that the more positive change from baseline in CBI in the fluoxetine group did not occur immediately, instead only becoming apparent during weeks 2 and 3 of treatment. This difference was no longer apparent following cessation of drug treatment. (C/D/E/F) Behavioural data were modelled using the diffusion model and parameters corresponding to the relative starting point (*zr*), drift rate (*v*) and boundary separation (*a*) were analysed. Data shown are averages for all probe sessions during that experimental period. (C) Although not significant, chronic antidepressant treatment showed a tendency to cause a more positive *zr*. This was both compared to the groups own baseline, and compared to controls. (D) Analysis of this measure by week indicated that *zr* was more positive compared to baseline in weeks 2 and 3, the same weeks that there was a more positive change in CBI. (E) There were no differences in *v* between the control and fluoxetine-treated groups. (F) There were also no changes in boundary separation (α). Change from baseline (average of the two pre-drug test sessions) measures were calculated to take into account individual differences in underlying bias. Data shown are for the midpoint tone only, and represent mean±SEM. Control group: *n*=9, fluoxetine-treated group: *n*=8. *p<0.05, ^#^*p*≤0.09.

Seventeen rats (*n*=9 control, *n*=8 fluoxetine) were included in the analysis. There were no differences between the control and fluoxetine group for any variables analysed in the two pre-drug probe test sessions (Figure 4D–F, Figure S4 and Table S4). Overall analysis of the entire study period (pre-drug, drug and post-drug) did not show any main effects on change in CBI, although there was a trend towards a group difference (*F*_1,15_=3.895, *p*=0.067), which when analysed excluding the pre-drug period (as measures for this period were matched between groups) indicated that the fluoxetine group become more positive relative to the control group during the treatment period only (main effect of session: *F*_4.82,72.31_=4.724, *p*=0.002, and significant group difference during sessions 3–8 (drug period): *F*_1,15_=6.642, *p*=0.021; Figure 4A). However, there were no significant differences for individual sessions during the treatment period in post-hoc comparisons. In the first probe test following cessation of treatment (4 days later; session 9 in the post-drug period of Figure 4A), this difference between groups was no longer apparent. A separate analysis of the drug period by week indicated that the fluoxetine group were not more positive than controls immediately following onset of antidepressant treatment, instead only becoming different in weeks 2 and 3 (independent samples *t*-tests: *p*s≤0.048; Figure 4B). Chronic fluoxetine treatment did not alter any other behavioural measure (Figure S4). Chronic antidepressant treatment did not significantly alter any diffusion model parameters in the overall analysis (Figure 4C/E/F and Table S4). However, analysing change in relative starting point by week showed that as for the behaviour, in weeks two and three this parameter became more positive in the fluoxetine group compared to the control group (independent sample *t*-tests: *p*s≤0.039; Figure 4D).

### 3.4 Acute effects of psychostimulants

**Figure 5:**
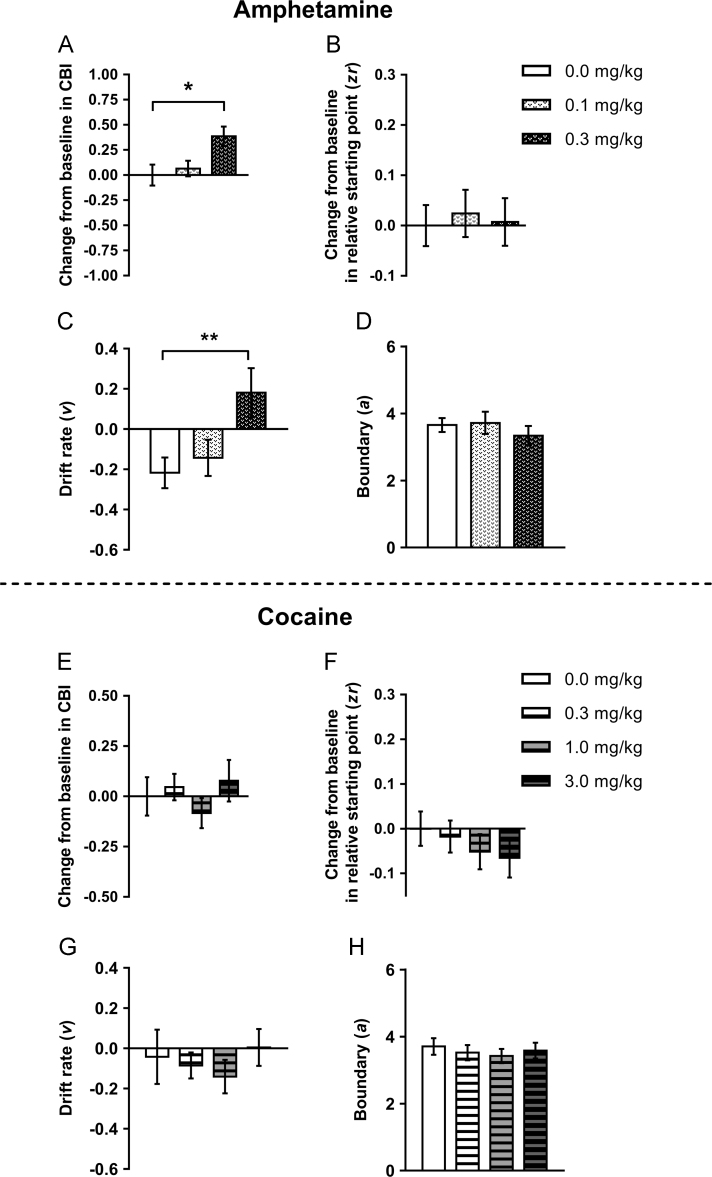
The effect of acute treatment with psychostimulants on behaviour and modelling of judgement bias. Amphetamine (0.1, 0.3 mg/kg) or cocaine (0.3, 1.0, 3.0 mg/kg) or saline vehicle (0.0 mg/kg) were administered acutely prior to testing on the judgement bias task. (A/E) Amphetamine (0.3 mg/kg) induced a positive judgement bias, whilst cocaine had no effect. (B/C/D) As with the positive bias induced acutely by ketamine, diffusion modelling indicated a more positive drift rate was also underlying the positive bias induced by amphetamine. Amphetamine had no effect on other model parameters. (F/G/H) Cocaine did not alter any diffusion model parameters. Data shown are for the midpoint tone only, and represent mean±SEM. Amphetamine: *n*=15, 15 min pre-treatment; cocaine: *n*=17, 10 min pre-treatment. **p<0.01.

For amphetamine, fifteen rats were included in the analysis. However, the highest dose (1.0 mg/kg) had to be excluded from analysis as rats completed insufficient trials. Amphetamine treatment (0.3 mg/kg) caused a positive judgement bias (main effect of session: *F*_1.535,21.485_=4.414, *p*=0.033 and post-hoc test: *p*=0.041; positive change from baseline in CBI; one-sample *t*-test: *p*=0.001; Figure 5A). This was also seen as an increase in percentage positive responding for the midpoint tone (Figure S5B). This dose caused an increase in omissions for the low reward tone (Figure S5C). Both doses of amphetamine caused an increase in premature responses (Figure S5D) but no changes in response latency (Figure S5A). The positive judgement bias was caused by a more positive drift rate (*v*) for 0.3 mg/kg compared to vehicle (main effect of session: *F*_2,28_=6.444, *p*=0.005 and pairwise comparison: *p*=0.008; Figure 5C), as no other diffusion model parameters were altered by amphetamine (Figure 5B/D and Table S4).

For the cocaine dose response, seventeen rats met criteria to be included in the analysis. There was no effect of any dose of cocaine (0.3, 1.0, 3.0 mg/kg) on any behavioural measure for the midpoint tone (Figure 5E and Figure S5E–H). Similarly, cocaine did not alter any diffusion model parameters (Figure 5F–H and Table S4) apart from 3.0 mg/kg cocaine caused the variability in the decision starting point to increase (Table S4).

## 4 Discussion

These experiments have revealed that delayed versus rapid onset antidepressant treatments exhibit temporally distinct effects on emotional decision making in rodents. The time course of the effects we observed are similar to the timescales associated with the subjective reporting of improved mood in patients. In the judgement bias task, affective bias was altered following chronic but not acute treatment with fluoxetine. In contrast, an immediate change was observed following acute administration of the NMDA antagonist, ketamine, and the psychostimulant, amphetamine. Another NMDA antagonist, PCP, had no effect, reflecting the lack of known antidepressant efficacy for this drug.

Previous studies have reported acute effects of ketamine in many rodent models of depression (reviewed by Browne and Lucki (2013)). However, antidepressant efficacy is also seen in many of these tests following acute treatment with conventional antidepressants. This contradicts the rate of onset of efficacy for these drugs. Patients with MDD only report subjective changes in mood weeks after beginning to take antidepressants (Anderson et al., 2000), with a meta-analysis of timing of onset of antidepressant efficacy finding that the majority of improvement was apparent after two weeks of treatment (Posternak and Zimmerman, 2005). Using the judgement bias task, we observed biases in the positive direction in decision making over timeframes that match the rapid versus delayed onset action of the drugs tested, i.e. immediately after a single acute treatment for ketamine, but only after two weeks for chronic treatment with fluoxetine. It must be noted that rats treated with ketamine did not become positive overall, only less negative than during vehicle treatment. In clinical trials, ketamine infusions in people rapidly reduce negative mood (e.g. Zarate et al., 2006) but it is not clear whether this treatment causes positive mood in patients or also makes them less negative. This is an area we hope to explore further in our future work using the judgement bias task.

Similarly, rats treated with chronic fluoxetine were not more positive relative to their own baseline, but rather only compared to the control group, where CBI was reduced during the treatment period. This suggests that chronic antidepressant treatment may not have been inducing a positive bias, but rather ameliorating a negative bias that could have been caused through the stress of repeated daily injections. However, even if this is the case it is clear that it is only chronic rather than acute antidepressant treatment where any effect on bias occurs.

The ketamine doses used in these studies were lower than those often reported to display antidepressant efficacy in the forced swim test, whilst for the conventional antidepressants tested, these doses are in line with ~70% receptor occupancy (Stuart et al., 2013). This task may therefore provide a novel and sensitive behavioural approach for detecting rate of onset of antidepressant treatment in a way which is relevant to how a drug will affect the subjective experience of mood in people.

The precise details of ketamine's mechanism of rapid antidepressant action are currently unclear, but our results suggest that this is not specific to NMDA receptor antagonism, as PCP failed to induce any effect. This is in keeping with clinical observations, where it has been shown that other NMDA receptor antagonists (e.g. memantine, lanicemine and MK-0657) do not reliably produce rapid-onset antidepressant action in patients with MDD (Newport et al., 2015). Zanos et al. (2016) have recently provided convincing evidence for the importance of sustained activation of AMPA receptors by the ketamine metabolite (2S,6S;2R,6R)-hydroxynorketamine. Other potential target pathways through which ketamine exerts rapid-onset antidepressant efficacy may include BDNF (brain-derived neurotrophic factor), mTOR (mammalian target of rapamycin) and GSK-3 (glycogen synthase kinase 3; (summarised by Drewniany et al. (2015)).

The data derived from diffusion modelling shows that rapid versus delayed onset antidepressants differ in their effects on decision making processes, which suggests different underlying neurobiology. A less negative drift rate was found to explain the positive bias induced acutely by ketamine whereas a more positive decision starting point was seen following chronic treatment with fluoxetine. Furthermore, despite a lack of effect on behaviour, the higher doses of reboxetine (1.0 mg/kg) and venlafaxine (3.0 mg/kg) also caused significant positive changes in the decision starting point. The opposite direction of the decision starting point and behaviour may seem contradictory, but diffusion model parameters are fit to both accuracy and RT behavioural data, not just CBI. This suggests that conventional antidepressant drugs act to alter the neuropsychological processes driving bias via a different mechanism to rapidly acting drugs. This is also consistent with findings from the rodent affective bias test, where venlafaxine and ketamine have differential effects on biases associated with learning and memory (Stuart et al., 2015). The ability of the diffusion model to detect this in both acute and chronic conventional antidepressant treatments, even where significant behavioural results are not detected, highlights the advantage of using the more sensitive diffusion modelling approach. Diffusion modelling results from this study are also consistent with a previous study where a more negative drift rate was found to underlie negative bias caused by an acute pharmacological treatment, whereas a more negative starting point explained negative bias induced over a longer time period by a chronic stress manipulation (Hales et al., 2016).

Treatment with two psychostimulant drugs – amphetamine and cocaine – reproduced previously published results from a reward versus avoidance of punishment version of this task (Rygula et al., 2014a, Rygula et al., 2014b). Amphetamine (0.3 mg/kg) induced a positive judgement bias, whilst cocaine had no effect. Although amphetamine and cocaine both affect monoamine levels in the brain, cocaine has a great selectively for the dopamine system whereas amphetamine affects noradrenaline, serotonin and dopamine (McMillen, 1983). It may be that this broader effect on monoamine levels in the brain contributes to the dissociation between these two treatments and judgement bias. The differences between the effects of amphetamine on judgement bias versus its lack of effect on affective biases linked with learning and memory (Stuart et al., 2013) further supports our hypothesis that this task is sensitive to treatments which induce subjective changes in mood in humans. As with the positive bias induced acutely by ketamine, diffusion modelling indicated a more positive drift rate was also underlying the positive bias induced by amphetamine. Interestingly, from the 1940s through to the mid-1960s, amphetamine was quite widely used for its antidepressant effects due to its ability to rapidly enhance mood and counteract the effects of anhedonia (Rasmussen, 2006). Both amphetamine and ketamine are psychotomimetic drugs, a common factor that may provide an alternative explanation for the positive changes in bias. However, PCP has similar properties but did not cause any change in bias, meaning this is unlikely to fully explain the effects on judgement bias observed. Like ketamine, amphetamine has no acute effect on affective biases linked with learning and memory (Stuart et al., 2013), further supporting our hypothesis that the judgement bias task is sensitive to treatments which induce subjective changes in mood in humans.

These findings suggest that the differential time course of effects seen in patients following treatment with delayed versus rapid onset antidepressants is replicated in the judgement bias task, suggesting this task has much better predictive validity than previous methods. Importantly, we observe effects with ketamine but not PCP, consistent with clinical observations. Taken together with previous studies, these data suggest the judgement bias task can better fulfil the criteria proposed for assessment of an animal model. The use of the modelling methods has also enabled us to gain more insight into the underlying cognitive processes which, going forward, may further add to the construct validity of the task.

Affective biases are observed across several different cognitive domains (Mathews and MacLeod, 2005, Clark et al., 2009, Gotlib and Joormann, 2010, Robinson and Roiser, 2016). Acute treatments with conventional antidepressants have been shown to positively bias emotional learning and memory and emotional interpretation in humans without having subjective effects on mood (Harmer et al., 2003, Harmer et al., 2009b, Harmer et al., 2013). In the rodent affective bias test (Stuart et al., 2013), acute treatment with conventional, delayed onset antidepressants also positively bias learning and memory whilst ketamine alone has no effect (Stuart et al., 2015). In the present study, acute treatments with conventional antidepressants did not induce positive biases in decision making, consistent with other work (Anderson et al., 2013, Rygula et al., 2014a). However, treatments that are known to induce rapid changes in mood, ketamine and amphetamine, did induce positive judgement biases in this rodent task, which can be dissociated from positive biases induced by drugs causing slower onset mood through the changes caused to diffusion model parameters. Taken together, these findings suggest that affective biases associated with different cognitive processes, as well as those induced by rapid versus delayed onset antidepressants, are likely to have different underlying neurobiology. The observation of positive judgement biases across timescales matching subjective changes in mood means that this task, which has been translated for use in humans (Paul et al., 2011, Anderson et al., 2012, Schick et al., 2013, Schick et al., 2015), may offer a valuable method to further investigate these differences.

## Role of the funding source

Funding for this study was provided by the Wellcome Trust Doctoral Training Programme in Neural Dynamics, Grant no. 099699/Z/12/Z. The Wellcome Trust had no further role in study design; in the collection, analysis and interpretation of data; in the writing of the report; and in the decision to submit the paper for publication.

## Contributors

C.A.H. conducted the experimental manipulations, collected and analysed the data, carried out modelling, and wrote the paper. All authors designed the research, discussed the analyses, results, and interpretation, revised the paper and approved the final manuscript.

## Conflict of interest

The authors declare no conflict of interest.

## Acknowledgements

C.J.H. is funded by James S. McDonnell Foundation through a Scholar Award in Cognition and the Elizabeth Blackwell Institute through a Senior Fellowship. E.S.J.R. is currently funded by the University of Bristol and has received research funding from MRC, BBSRC, RCUK, Wellcome Trust and the British Pharmacological Society Integrative Pharmacology Fund.

## References

[bib1] Anderson I.M., Nutt D.J., Deakin J.F.W. (2000). Evidence-based guidelines for treating depressive disorders with antidepressants: a revision of the 1993 British Association for Psychopharmacology guidelines. J. Psychopharmacol..

[bib2] Anderson M., Hardcastle C., Munafò M., Robinson E.J. (2012). Evaluation of a novel translational task for assessing emotional biases in different species. Cogn. Affect. Behav. Neurosci..

[bib3] Anderson M., Munafò M., Robinson E.J. (2013). Investigating the psychopharmacology of cognitive affective bias in rats using an affective tone discrimination task. Psychopharmacology.

[bib4] Beck A.T. (1976). Cognitive Therapy and the Emotional Disorders.

[bib5] Beck A.T. (2008). The evolution of the cognitive model of depression and its neurobiological correlates. Am. J. Psychiatry.

[bib6] Belzung C., Lemoine M. (2011). Criteria of validity for animal models of psychiatric disorders: focus on anxiety disorders and depression. Biol. Mood Anxiety Disord..

[bib7] Benn A., Robinson E.S. (2014). Investigating glutamatergic mechanism in attention and impulse control using rats in a modified 5-choice serial reaction time task. PLoS One.

[bib8] Berton O., Hahn C.G., Thase M.E. (2012). Are we getting closer to valid translational models for major depression?. Science.

[bib9] Browne C.A., Lucki I. (2013). Antidepressant effects of ketamine: mechanisms underlying fast-acting novel antidepressants. Front. Pharmacol..

[bib10] Butler G., Mathews A. (1983). Cognitive processes in anxiety. Adv. Behav. Res. Ther..

[bib11] Clark L., Chamberlain S.R., Sahakian B.J. (2009). Neurocognitive mechanisms in depression: implications for treatment. Annu. Rev. Neurosci..

[bib12] Cryan J.F., Holmes A. (2005). The ascent of mouse: advances in modelling human depression and anxiety. Nat. Rev. Drug Discov..

[bib13] Cryan J.F., Slattery D.A. (2007). Animal models of mood disorders: recent developments. Curr. Opin. Psychiatry.

[bib14] Drewniany E., Han J., Hancock C., Jones R.L., Lim J., Nemat Gorgani N., Sperry J.K., Yu H.J., Raffa R.B. (2015). Rapid-onset antidepressant action of ketamine: potential revolution in understanding and future pharmacologic treatment of depression. J. Clin. Pharm. Ther..

[bib15] Duman R.S., Li N. (2012). A neurotrophic hypothesis of depression: role of synaptogenesis in the actions of NMDA receptor antagonists. Philos. Trans. R. Soc. Lond. B Biol. Sci..

[bib16] Duman R.S., Li N., Liu R.J., Duric V., Aghajanian G. (2012). Signaling pathways underlying the rapid antidepressant actions of ketamine. Neuropharmacology.

[bib17] Duman R.S., Monteggia L.M. (2006). A neurotrophic model for stress-related mood disorders. Biol. Psychiatry.

[bib18] Duman R.S., Voleti B. (2012). Signaling pathways underlying the pathophysiology and treatment of depression: novel mechanisms for rapid-acting agents. Trends Neurosci..

[bib19] Gotlib I.H., Joormann J. (2010). Cognition and depression: current status and future directions. Ann. Rev. Clin. Psychol..

[bib20] Hales C.A., Robinson E.S., Houghton C.J. (2016). Diffusion Modelling reveals the decision making processes underlying negative judgement bias in rats. PLoS One.

[bib21] Hales C.A., Stuart S.A., Anderson M.H., Robinson E.S.J. (2014). Modelling cognitive affective biases in major depressive disorder using rodents. Br. J. Pharmacol..

[bib22] Harding E.J., Paul E.S., Mendl M. (2004). Animal behaviour: cognitive bias and affective state. Nature.

[bib23] Harmer C.J. (2010). Antidepressant drug action: a neuropsychological perspective. Depress. Anxiety.

[bib24] Harmer C.J., Bhagwagar Z., Perrett D.I., Vollm B.A., Cowen P.J., Goodwin G.M. (2003). Acute SSRI administration affects the processing of social cues in healthy volunteers. Neuropsychopharmacology.

[bib25] Harmer C.J., Dawson G.R., Dourish C.T., Favaron E., Parsons E., Fiore M., Zucchetto M., Bifone A., Poggesi I., Fernandes S., Alexander R.C., Goodwin G.M. (2013). Combined NK(1) antagonism and serotonin reuptake inhibition: effects on emotional processing in humans. J. Psychopharmacol..

[bib26] Harmer C.J., de Bodinat C., Dawson G.R., Dourish C.T., Waldenmaier L., Adams S., Cowen P.J., Goodwin G.M. (2011). Agomelatine facilitates positive versus negative affective processing in healthy volunteer models. J. Psychopharmacol..

[bib27] Harmer C.J., Goodwin G.M., Cowen P.J. (2009). Why do antidepressants take so long to work? A cognitive neuropsychological model of antidepressant drug action. Br. J. Psychiatry.

[bib28] Harmer C.J., O'Sullivan U., Favaron E., Massey-Chase R., Ayres R., Reinecke A., Goodwin G.M., Cowen P.J. (2009). Effect of acute antidepressant administration on negative affective bias in depressed patients. Am. J. Psychiatry.

[bib29] Harmer C.J., Shelley N.C., Cowen P.J., Goodwin G.M. (2004). Increased positive versus negative affective perception and memory in healthy volunteers following selective serotonin and norepinephrine reuptake inhibition. Am. J. Psychiatry.

[bib30] Kircanski K., Joormann J., Gotlib I.H. (2012). Cognitive aspects of depression. Wiley Interdiscip. Rev. Cogn. Sci..

[bib31] Mathews A., MacLeod C. (2005). Cognitive vulnerability to emotional disorders. Annu. Rev. Clin. Psychol..

[bib32] McMillen B.A. (1983). CNS stimulants: two distinct mechanisms of action for amphetamine-like drugs. Trends Pharmacol. Sci..

[bib33] Murphy F.C., Sahakian B.J., Rubinsztein J.S., Michael A., Rogers R.D., Robbins T.W., Paykel E.S. (1999). Emotional bias and inhibitory control processes in mania and depression. Psychol. Med..

[bib34] Nestler E.J., Gould E., Manji H., Buncan M., Duman R.S., Greshenfeld H.K., Hen R., Koester S., Lederhendler I., Meaney M., Robbins T., Winsky L., Zalcman S. (2002). Preclinical models: status of basic research in depression. Biol. Psychiatry.

[bib35] Nestler E.J., Hyman S.E. (2010). Animal models of neuropsychiatric disorders. Nat. Neurosci..

[bib36] Newport D.J., Carpenter L.L., McDonald W.M., Potash J.B., Tohen M., Nemeroff C.B. (2015). Ketamine and other NMDA antagonists: early clinical trials and possible mechanisms in depression. Am. J. Psychiatry.

[bib37] Nunn J.D., Mathews A., Trower P. (1997). Selective processing of concern-related information in depression. Br. J. Clin. Psychol..

[bib38] O'Leary O.F., Cryan J.F. (2013). Towards translational rodent models of depression. Cell Tissue Res..

[bib39] Papciak J., Popik P., Fuchs E., Rygula R. (2013). Chronic psychosocial stress makes rats more ‘pessimistic’ in the ambiguous-cue interpretation paradigm. Behav. Brain Res..

[bib40] Paul E.S., Cuthill I., Kuroso G., Norton V., Woodgate J., Mendl M. (2011). Mood and the speed of decisions about anticipated resources and hazards. Evol. Hum. Behav..

[bib41] Porsolt R.D., Le Pichon M., Jalfre M. (1977). Depression: a new animal model sensitive to antidepressant treatments. Nature.

[bib42] Posternak M.A., Zimmerman M. (2005). Is there a delay in the antidepressant effect? A meta-analysis. J. Clin. Psychiatry.

[bib43] Rasmussen N. (2006). Making the first anti-depressant: amphetamine in American medicine, 1929–1950. J. Hist. Med. Allied Sci..

[bib44] Ratcliff R. (1978). A theory of memory retrieval. Psychol. Rev..

[bib45] Ratcliff R., Rouder J.N. (1998). Modeling response times for two-choice decisions. Psychol. Sci..

[bib46] Robinson E.S., Roiser J.P. (2016). Affective biases in humans and animals. Curr. Top. Behav. Neurosci..

[bib47] Robinson O.J., Letkiewicz A.M., Overstreet C., Ernst M., Grillon C. (2011). The effect of induced anxiety on cognition: threat of shock enhances aversive processing in healthy individuals. Cogn. Affect. Behav. Neurosci..

[bib48] Roiser J.P., Elliott R., Sahakian B.J. (2012). Cognitive mechanisms of treatment in depression. Neuropsychopharmacology.

[bib49] Rygula R., Papciak J., Popik P. (2013). Trait pessimism predicts vulnerability to stress-induced anhedonia in rats. Neuropsychopharmacology.

[bib50] Rygula R., Papciak J., Popik P. (2014). The effects of acute pharmacological stimulation of the 5-HT, NA and DA systems on the cognitive judgement bias of rats in the ambiguous-cue interpretation paradigm. Eur. Neuropsychopharmacol..

[bib51] Rygula R., Szczech E., Papciak J., Nikiforuk A., Popik P. (2014). The effects of cocaine and mazindol on the cognitive judgement bias of rats in the ambiguous-cue interpretation paradigm. Behav. Brain Res..

[bib52] Schick A., Adam R., Vollmayr B., Kuehner C., Kanske P., Wessa M. (2015). Neural correlates of valence generalization in an affective conditioning paradigm. Behav. Brain Res..

[bib53] Schick A., Wessa M., Vollmayr B., Kuehner C., Kanske P. (2013). Indirect assessment of an interpretation bias in humans: neurophysiological and behavioral correlates. Front. Hum. Neurosci..

[bib54] Stuart S.A., Butler P., Munafo M.R., Nutt D.J., Robinson E.S. (2013). A translational rodent assay of affective biases in depression and antidepressant therapy. Neuropsychopharmacology.

[bib55] Stuart S.A., Butler P., Munafo M.R., Nutt D.J., Robinson E.S. (2015). Distinct neuropsychological mechanisms may explain delayed- versus rapid-onset antidepressant efficacy. Neuropsychopharmacology.

[bib56] Stuart S.A., Robinson E.S.J. (2015). Reducing the stress of drug administration: implications for the 3Rs. Sci. Rep..

[bib57] Teasdale J.D. (1983). Negative thinking in depression: cause, effect, or reciprocal relationship?. Adv. Behav. Res. Ther..

[bib58] Teasdale J.D. (1988). Cognitive vulnerability to persistent depression. Cogn. Emot..

[bib59] Voncken M.J., Bogels S.M., Peeters F. (2007). Specificity of interpretation and judgemental biases in social phobia versus depression. Psychol. Psychother..

[bib60] Voss A., Nagler M., Lerche V. (2013). Diffusion models in experimental psychology: a practical introduction. J. Exp. Psychol..

[bib61] Voss A., Rothermund K., Gast A., Wentura D. (2013). Cognitive processes in associative and categorical priming: a diffusion model analysis. J. Exp. Psychol.-Gen..

[bib62] Voss A., Rothermund K., Voss J. (2004). Interpreting the parameters of the diffusion model: an empirical validation. Mem. Cogn..

[bib63] Voss A., Voss J. (2007). Fast-dm: a free program for efficient diffusion model analysis. Behav. Res. Methods.

[bib64] Voss A., Voss J. (2008). A fast numerical algorithm for the estimation of diffusion model parameters. J. Math. Psychol..

[bib65] Voss A., Voss J., Klauer K.C. (2010). Separating response-execution bias from decision bias: arguments for an additional parameter in Ratcliff's diffusion model. Br. J. Math. Stat. Psychol..

[bib66] Voss A., Voss J., Lerche V. (2015). Assessing cognitive processes with diffusion model analyses: a tutorial based on fast-dm-30. Front. Psychol..

[bib67] Wang J., Jing L., Toledo-Salas J.C., Xu L. (2015). Rapid-onset antidepressant efficacy of glutamatergic system modulators: the neural plasticity hypothesis of depression. Neurosci. Bull..

[bib68] White C., Ratcliff R., Vasey M., McKoon G. (2009). Dysphoria and memory for emotional material: a diffusion-model analysis. Cogn. Emot..

[bib69] White C.N., Ratcliff R., Vasey M.W., McKoon G. (2010). Anxiety enhances threat processing without competition among multiple inputs: a diffusion model analysis. Emotion.

[bib70] Willner P. (1984). The validity of animal models of depression. Psychopharmacol.

[bib71] Willner P. (2005). Chronic mild stress (CMS) revisited: consistency and behavioural-neurobiological concordance in the effects of CMS. Neuropsychobiology.

[bib72] Zanos P., Moaddel R., Morris P.J., Georgiou P., Fischell J., Elmer G.I., Alkondon M., Yuan P., Pribut H.J., Singh N.S., Dossou K.S., Fang Y., Huang X.P., Mayo C.L., Wainer I.W., Albuquerque E.X., Thompson S.M., Thomas C.J., Zarate C.A., Gould T.D. (2016). NMDAR inhibition-independent antidepressant actions of ketamine metabolites. Nature.

[bib73] Zarate C.A., Singh J.B., Carlson P.J., Brutsche N.E., Ameli R., Luckenbaugh D.A., Charney D.S., Manji H.K. (2006). A randomized trial of an N-methyl-D-aspartate antagonist in treatment-resistant major depression. Arch. Gen. Psychiatry.

