# Supplementary material for: Behavioural and computational methods reveal differential effects for how delayed and rapid onset antidepressants effect decision making in rats
Source: Eur Neuropsychopharmacol. 2017 Dec;27(12):1268–80. doi: 10.1016/j.euroneuro.2017.09.008 (PMC5720479; doi:10.1016/j.euroneuro.2017.09.008)
Supplement: Supplementary file 1 — Supplementary material [file mmc1.docx]

# Supplementary Materials and Methods

## Apparatus

Operant chambers (30.5 x 24.1 x 21.0 cm) used for behavioural testing were housed inside a light-resistant and sound-attenuating box. They were equipped with two retractable response levers positioned on each side of the centrally located food magazine. The magazine had a house light (28 V, 100 mA) located above it. An audio generator (ANL-926, Med Associates, Sandown Scientific, UK) produced tones that were delivered to each chamber via a speaker positioned above the left lever. Operant chambers and audio generators were controlled using K-Limbic software (Conclusive Solutions Ltd., UK). The midpoint ambiguous probe tone was chosen to be equidistant from the reference tones based on the mean frequency difference limen in hooded rats (Syka et al., 1996), and tone loudness was adjusted according to the hooded rat audiogram (Heffner et al., 1994).

## Behavioural task

Training stages and criteria are detailed in Supplementary Table S1. Training sessions consisted of 100 trials. Unless otherwise specified in Table S1, response levers were extended at the beginning of every session and remained extended for the duration of the session (maximum one hour for all session types). During sessions pressing the incorrect lever during a tone was punished by a 5 s timeout, as was an omission if the rat failed to press any lever during the 20 s tone. Tone presentations were separated by an inter-trial interval of 5 s, during which time premature responses on either lever were punished by a 20 s timeout. During a timeout, the house light was illuminated, and responses made on levers were recorded but had no programmed consequences. In order to allow for random reinforcement with constraints of the software, the ambiguous midpoint tone was made up of one of two tones (20 x 4,999 Hz and 20 x 5,001 Hz at 70 dB). This meant that the outcome associated with each of the two ambiguous tones could be programmed to be the same as the reference tone they were closer to, hence resulting in random reinforcement. Responses to either of the two midpoint tones were analysed together.

## Modelling

Behavioural data entered into the model were tone, response latency and which lever (high or low reward) the response was made on (corresponding to whether the decision process reached the upper or lower boundary respectively). Fast-dm-30 calculates predictive cumulative distribution functions for behavioural RTs and uses this to fit diffusion model parameters. For this task, parameters corresponding to decision starting point (*zr*), drift rate (*v*), and boundary separation (*a*) were fit to RTs from each tone, while non-decision RT (*t*_0_), inter-trial variability in the starting point (*szr*) and difference in speed of execution between the two responses (*d*) were fit to data from all tones combined. The two other parameters that can be fit by the diffusion model (*sv* and *st*_0_) were set to zero. Trials with short response latencies (<200 ms) were removed from the analysis as in previous studies (White et al., 2009, 2010, 2011; Hales et al., 2016), as it is considered that these do not reflect a true decision making accumulation process in response to the cue (Vandekerckhove & Tuerlinckx, 2007).

Supplementary References

Heffner HE, Heffner RS, Contos C, Ott T (1994). Audiogram of the hooded Norway rat. *Hearing Research* 73(2): 244-247.

Syka J, Rybalko N, Brožek G, Jilek M (1996). Auditory frequency and intensity discrimination in pigmented rats. *Hearing Research* 100(1): 107-113.

Vandekerckhove J, Tuerlinckx F (2007). Fitting the Ratcliff diffusion model to experimental data. *Psychonomic Bulletin & Review* 14(6): 1011-1026.

White C, Ratcliff R, Vasey M, McKoon G (2009). Dysphoria and memory for emotional material: A diffusion-model analysis. *Cognition and Emotion* 23(1): 181-205.

White CN, Ratcliff R, Starns JJ (2011). Diffusion models of the flanker task: Discrete versus gradual attentional selection. *Cognitive Psychology* 63(4): 210-238.

White CN, Ratcliff R, Vasey MW, McKoon G (2010). Anxiety enhances threat processing without competition among multiple inputs: A diffusion model analysis. *Emotion* 10(5): 662-677.
