# Supplementary material for: Behavioural and computational methods reveal differential effects for how delayed and rapid onset antidepressants effect decision making in rats
Source: Eur Neuropsychopharmacol. 2017 Dec;27(12):1268–80. doi: 10.1016/j.euroneuro.2017.09.008 (PMC5720479; doi:10.1016/j.euroneuro.2017.09.008)
Supplement: Supplementary file 2 — Supplementary material [file mmc2.docx]

Table S1 - Training stages and required performance criteria for the judgement bias task.

| Stage | Description | Criteria |
| --- | --- | --- |
| **1 –**  **Magazine**  **training** | Tone (high or low only on alternate sessions) played for 5 s followed by release of one pellet into magazine; 30 s ITI. No levers available. | All sugar pellets eaten |
| **2 –**  **Lever**  **training** | Response on lever during tone (high or low only on alternate sessions) rewarded with one pellet. Only correct lever available. | > 70 responses for two consecutive sessions |
| **3 –**  **Tone**  **training** | Response on correct corresponding lever only during tone (high or low only on alternate sessions) rewarded with one pellet. Both levers available. | > 70% accuracy and  < 1:1 ratio of correct:premature responses on two consecutive sessions |
| **4 – Discrimination**  **training** | Response on correct corresponding lever only during tone (either high or low presented pseudorandomly) rewarded with one pellet. Both levers available. | > 70% accuracy for both tones, no significant differences on analysed behavioural measures over three sessions and < 1:1 ratio of correct:premature responses |
| **5 –**  **Reward**  **magnitude**  **training** | As Stage 4 but response on correct corresponding lever only rewarded with four pellets for high reward tone and one pellet for low reward tone. Both levers available. | As for Stage 4 but with > 60% accuracy for both tones  (to allow for biases in responding to reference tones caused by the difference in associated reward magnitude) |

Training sessions consisted of 100 trials. Apart from where otherwise specified in the description, for all sessions (training, baseline and probe test sessions), response levers were extended at the beginning of every session and remained extended for the duration of the session (maximum one hour). During sessions pressing the incorrect lever during a tone was punished by a 5 s timeout, as was an omission if the rat failed to press any lever during the 20 s tone. Tone presentations were separated by an inter-trial interval of 5 s, during which time premature responses on either lever were punished by a 20 s timeout. During a timeout, the house light was illuminated, and responses made on levers were recorded but had no programmed consequences.
