# Supplementary material for: Behavioural and computational methods reveal differential effects for how delayed and rapid onset antidepressants effect decision making in rats
Source: Eur Neuropsychopharmacol. 2017 Dec;27(12):1268–80. doi: 10.1016/j.euroneuro.2017.09.008 (PMC5720479; doi:10.1016/j.euroneuro.2017.09.008)
Supplement: Supplementary file 3 — Supplementary material [file mmc3.docx]

Table S2 – Experimental design for acute drug studies

| Experiment | Cohort | No. of treatments | Drugs and doses | Treatment order | n = | |
| --- | --- | --- | --- | --- | --- | --- |
|  |  |  |  |  | **Dosed** | **Included in analyses** |
| Acute effects of conventional, delayed onset antidepressants | 1 | 7 | 0.0 mg/kg (vehicle)  0.3 mg/kg, 1.0 mg/kg fluoxetine  0.3 mg/kg, 1.0 mg/kg reboxetine  1.0 mg/kg, 3.0 mg/kg venlafaxine | - | 14 | 9 |
| Acute effects of NMDA receptor antagonists | 2 | 4 | 0.0 mg/kg (vehicle)  0.3, 1.0, 3.0 mg/kg ketamine | 1 | 18 | 13 |
|  | 2 | 4 | 0.0 mg/kg (vehicle)  0.3, 1.0, 3.0 mg/kg PCP | 4 | 18 | 16 |
|  | 3 | 2 | 1. mg/kg (vehicle) 2. mg/kg ketamine | - | 16 | 15 |
| Acute effects of psychostimulants | 2 | 4 | 0.0 mg/kg (vehicle)  0.1, 0.3, 1.0 mg/kg amphetamine  (but only 0.1, 0.3 mg/kg included in analyses) | 2 | 18 | 15 |
|  | 2 | 4 | 0.0 mg/kg (vehicle)  0.3, 1.0, 3.0 mg/kg cocaine | 3 | 18 | 17 |

This table details the drugs and doses used for each within-subjects acute dose-response study. Drug doses within each study (row) were given in a full counterbalanced order. Drug treatments in cohort 2 (acute effects of NMDA receptor antagonists and acute effects of psychostimulants) were given in the order shown in the “Treatment order” column. PCP - phencyclidine.
