# Supplementary material for: Behavioural and computational methods reveal differential effects for how delayed and rapid onset antidepressants effect decision making in rats
Source: Eur Neuropsychopharmacol. 2017 Dec;27(12):1268–80. doi: 10.1016/j.euroneuro.2017.09.008 (PMC5720479; doi:10.1016/j.euroneuro.2017.09.008)
Supplement: Supplementary file 4 — Supplementary material [file mmc4.docx]

Table S3 – Description of statistical analysis for other behavioural measures

| Behavioural measure | Analysed for: | Description | Statistical analysis | |
| --- | --- | --- | --- | --- |
|  |  |  | **Acute dose response studies** | **Chronic fluoxetine study** |
| Response latency | Each tone | Time between presentation of the tone and response on the lever (correct lever for high and low reward tones, either lever for midpoint tone) | Two-way repeated measures ANOVA with tone and session as within-subjects factors | Mixed ANOVA with two repeated measures (tone and session) as the within-subjects factors and group as the between-subjects factor |
| Percentage positive responses | Each tone | Number of responses made on the high reward lever divided by the total number of responses made and on the high reward and low reward levers for that tone |  |  |
| Percentage omissions | Each tone | Number of trials where no lever press occurred during 20 s tone presentation divided by total completed trials for that tone |  |  |
| Percentage of premature responses | Whole session | Number of trials where a response was made in the 5 s inter-trial interval divided by total completed trials plus total premature trials | Repeated measures ANOVA with session as the within-subjects factor | Mixed ANOVA with session as the within-subjects factor and group as the between-subjects factor |

This table details the other behavioural measures (apart from cognitive bias index) that were analysed for each experimental manipulation and are presented in the supplementary figures. ANOVA – analysis of variance.
