# Supplementary material for: Behavioural and computational methods reveal differential effects for how delayed and rapid onset antidepressants effect decision making in rats
Source: Eur Neuropsychopharmacol. 2017 Dec;27(12):1268–80. doi: 10.1016/j.euroneuro.2017.09.008 (PMC5720479; doi:10.1016/j.euroneuro.2017.09.008)
Supplement: Supplementary file 5 — Supplementary material [file mmc5.docx]

| **Manipulation** | **Session / period** | ***t_0_*** | | ***szr*** | | ***d*** |
| --- | --- | --- | --- | --- | --- | --- |
| **Acute antidepressants** | 0.0 mg/kg | 0.439 ± 0.129 | 0.266 ± 0.068 | | 0.022 ± 0.015 | |
|  | Flu 0.3 mg/kg | 0.355 ± 0.094 | 0.264 ± 0.022 | | 0.022 ± 0.018 | |
|  | Flu 1.0 mg/kg | 0.440 ± 0.085 | 0.327 ± 0.052 | | 0.014 ± 0.022 | |
|  | Reb 0.3 mg/kg | 0.651 ± 0.281 | 0.286 ± 0.036 | | 0.034 ± 0.031 | |
|  | Reb 1.0 mg/kg | 1.018 ± 0.450 | **0.175 ± 0.031*** | | 0.009 ± 0.012 | |
|  | Ven 1.0 mg/kg | 0.419 ± 0.091 | 0.239 ± 0.030 | | 0.048 ± 0.022 | |
|  | Ven 3.0 mg/kg | 0.476 ± 0.113 | 0.298 ± 0.052 | | 0.024 ± 0.007 | |
| **Ketamine** | 0.0 mg/kg | 0.371 ± 0.054 | 0.303 ± 0.037 | | 0.007 ± 0.013 | |
|  | 0.3 mg/kg | 0.292 ± 0.062 | 0.270 ± 0.026 | | 0.020 ± 0.027 | |
|  | 1.0 mg/kg | 0.328 ± 0.059 | 0.353 ± 0.052 | | 0.016 ± 0.014 | |
|  | 3.0 mg/kg | 0.310 ± 0.054 | 0.305 ± 0.043 | | 0.020 ± 0.025 | |
| **PCP** | 0.0 mg/kg | 0.218 ± 0.047 | 0.214 ± 0.029 | | 0.019 ± 0.011 | |
|  | 0.3 mg/kg | 0.296 ± 0.050 | 0.285 ± 0.033 | | 0.008 ± 0.008 | |
|  | 1.0 mg/kg | 0.261 ± 0.044 | 0.300 ± 0.031 | | 0.018 ± 0.013 | |
|  | 3.0 mg/kg | **0.439 ± 0.076*** | 0.280 ± 0.036 | | 0.002 ± 0.009 | |
| **Chronic fluoxetine** (control group) | Pre | 0.258 ± 0.051 | 0.344 ± 0.034 | | 0.006 ± 0.016 | |
|  | Drug | 0.259 ± 0.036 | 0.312 ± 0.023 | | 0.020 ± 0.007 | |
|  | Post | 0.259 ± 0.049 | 0.308 ± 0.032 | | 0.024 ± 0.013 | |
| **Chronic fluoxetine** (fluoxetine group) | Pre | 0.279 ± 0.048 | 0.269 ± 0.028 | | 0.001 ± 0.007 | |
|  | Drug | 0.276 ± 0.039 | 0.280 ± 0.021 | | 0.015 ± 0.008 | |
|  | Post | 0.267 ± 0.052 | 0.236 ± 0.038 | | 0.010 ± 0.011 | |
| **Amphetamine** | 0.0 mg/kg | 0.312 ± 0.057 | 0.265 ± 0.030 | | 0.011 ± 0.012 | |
|  | 0.1 mg/kg | 0.281 ± 0.040 | 0.298 ± 0.033 | | 0.011 ± 0.007 | |
|  | 0.3 mg/kg | 0.246 ± 0.047 | 0.327 ± 0.029 | | -0.003 ± 0.016 | |
| **Cocaine** | 0.0 mg/kg | 0.266 ± 0.038 | 0.279 ± 0.023 | | 0.002 ± 0.007 | |
|  | 0.3 mg/kg | 0.315 ± 0.046 | 0.270 ± 0.009 | | 0.012 ± 0.010 | |
|  | 1.0 mg/kg | 0.214 ± 0.044 | 0.301 ± 0.028 | | 0.016 ± 0.009 | |
|  | 3.0 mg/kg | 0.376 ± 0.065 | **0.370 ± 0.034*** | | 0.011 ± 0.010 | |

Table S4 - Values for diffusion model parameters fit to all data for all experimental manipulations.

Values for three parameters (*t_0_*, *szr* and *d*) that were fit by the diffusion model using behavioural data from all tones are shown as mean ± SEM for each experimental manipulation. Data points highlighted in bold and denoted with * show where a significant difference was found for that parameter compared to the control/vehicle session. Flu – fluoxetine; Reb – reboxetine; Ven – venlafaxine; PCP - phencyclidine.
