# Supplementary material for: Behavioural and computational methods reveal differential effects for how delayed and rapid onset antidepressants effect decision making in rats
Source: Eur Neuropsychopharmacol. 2017 Dec;27(12):1268–80. doi: 10.1016/j.euroneuro.2017.09.008 (PMC5720479; doi:10.1016/j.euroneuro.2017.09.008)
Supplement: Supplementary file 6 — Supplementary material [file mmc6.pdf]

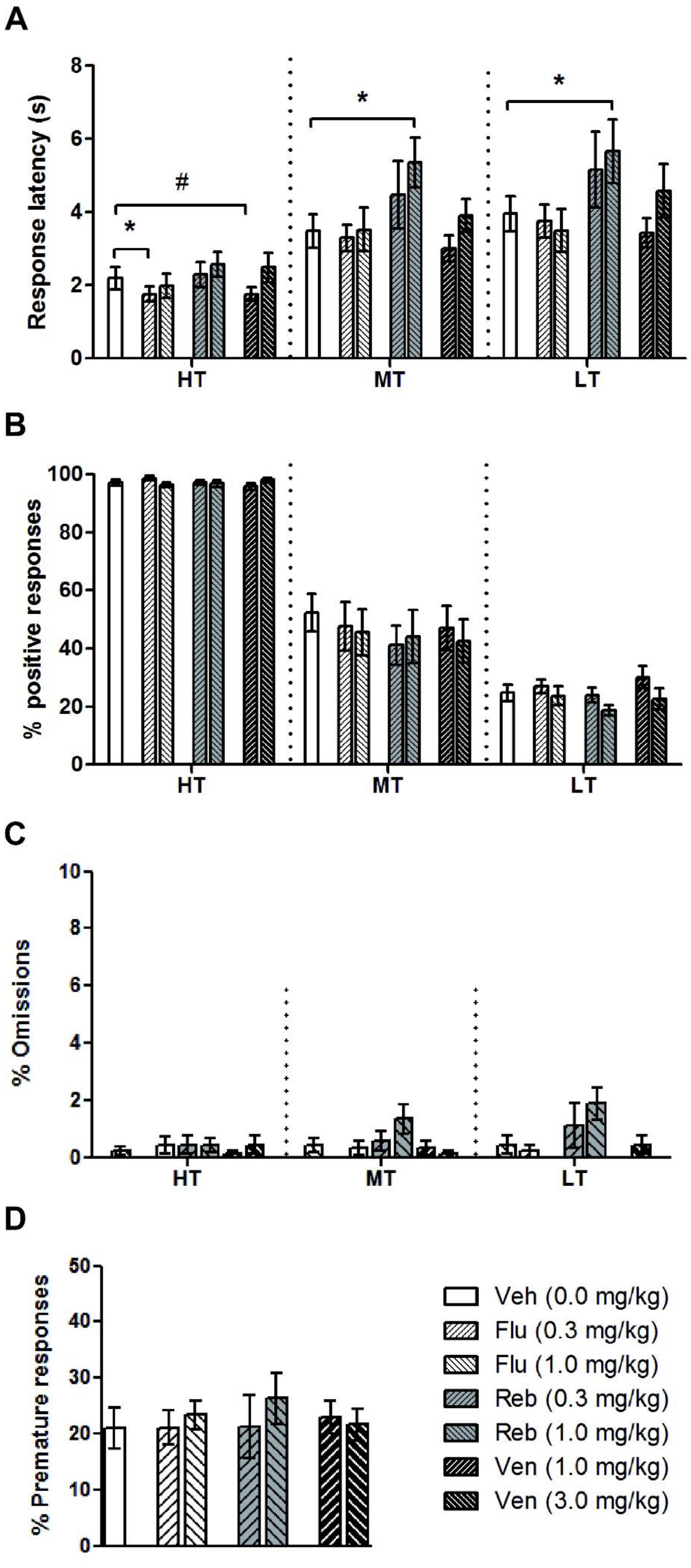

Supplementary Figure 1 – *Behavioural data from the judgement bias task following acute treatment with three antidepressants*. Acute doses of fluoxetine (0.3, 1.0 mg/kg), reboxetine (0.3, 1.0 mg/kg) or venlafaxine (1.0, 3.0 mg/kg) were administered by intraperitoneal injection to measure their effect on judgement bias. (A) The lower dose of fluoxetine (0.3 mg/kg) reduced response latency for the high reward tone (significant session*tone interaction: $F_{3.52,28.18}$=5.597, p=0.003 and post-hoc tests: p=0.013), with the lower dose of venlafaxine (1.0 mg/kg) tending towards the same effect (post-hoc test: p=0.067). The higher dose of reboxetine (1.0 mg/kg) significantly increased response latency for the midpoint and low reward tones (significant post-hoc tests: ps≤0.038). (B/C/D) There were no effects of any of the antidepressant drugs on the percentage of positive responses, percentage of omissions or percentage of premature responses. Data represent mean ± SEM. n=9, 30 min pre-treatment. *p<0.05, $^{\#}p$<0.07. Veh – vehicle; Flu – fluoxetine; Reb – reboxetine; Ven – venlafaxine; HT - high reward tone; MT - midpoint tone; LT - low reward tone.
