# Supplementary material for: Behavioural and computational methods reveal differential effects for how delayed and rapid onset antidepressants effect decision making in rats
Source: Eur Neuropsychopharmacol. 2017 Dec;27(12):1268–80. doi: 10.1016/j.euroneuro.2017.09.008 (PMC5720479; doi:10.1016/j.euroneuro.2017.09.008)
Supplement: Supplementary file 7 — Supplementary material [file mmc7.pdf]

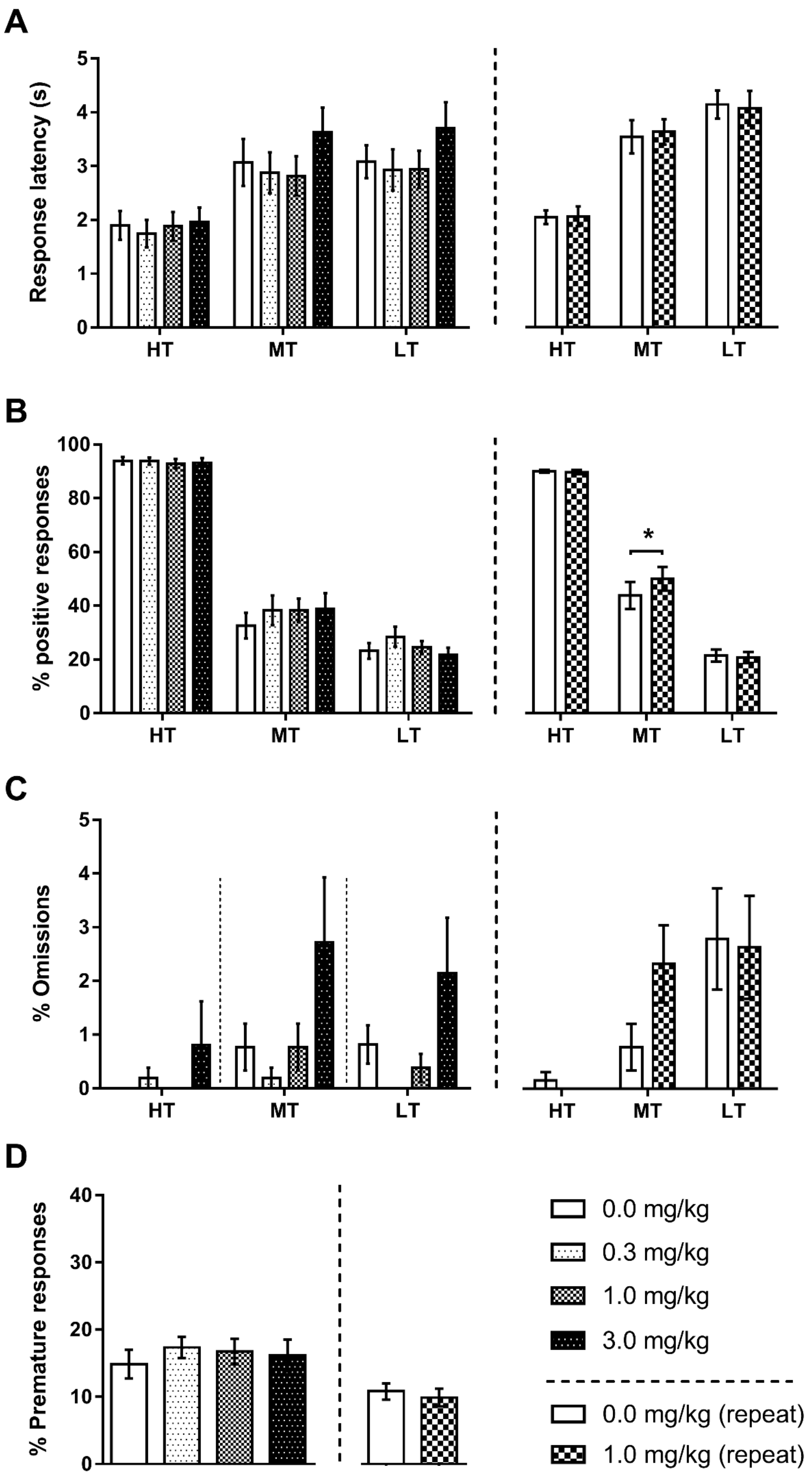

Supplementary Figure 2 – *Behavioural data from the judgement bias task following acute treatment with ketamine.* Acute doses of ketamine (initial study: 0.0, 0.3, 1.0, 3.0 mg/kg; replication study: 0.0, 1.0 mg/kg) were administered by intraperitoneal injection to measure their effect on judgement bias. (A/C/D) Ketamine had no effect on response latency, percentage omissions or percentage of premature responses in either the initial or replication study. (B) There was no overall main effect of ketamine on percentage of positive responses in the initial study, but in the replication study ketamine caused positive responding to increase for the midpoint tone. Data represent mean ± SEM. Initial study: n = 13; replication study: n = 15, 60 min pre-treatment. HT -high reward tone; MT -midpoint tone; LT -low reward tone.
