# Supplementary material for: Behavioural and computational methods reveal differential effects for how delayed and rapid onset antidepressants effect decision making in rats
Source: Eur Neuropsychopharmacol. 2017 Dec;27(12):1268–80. doi: 10.1016/j.euroneuro.2017.09.008 (PMC5720479; doi:10.1016/j.euroneuro.2017.09.008)
Supplement: Supplementary file 8 — Supplementary material [file mmc8.pdf]

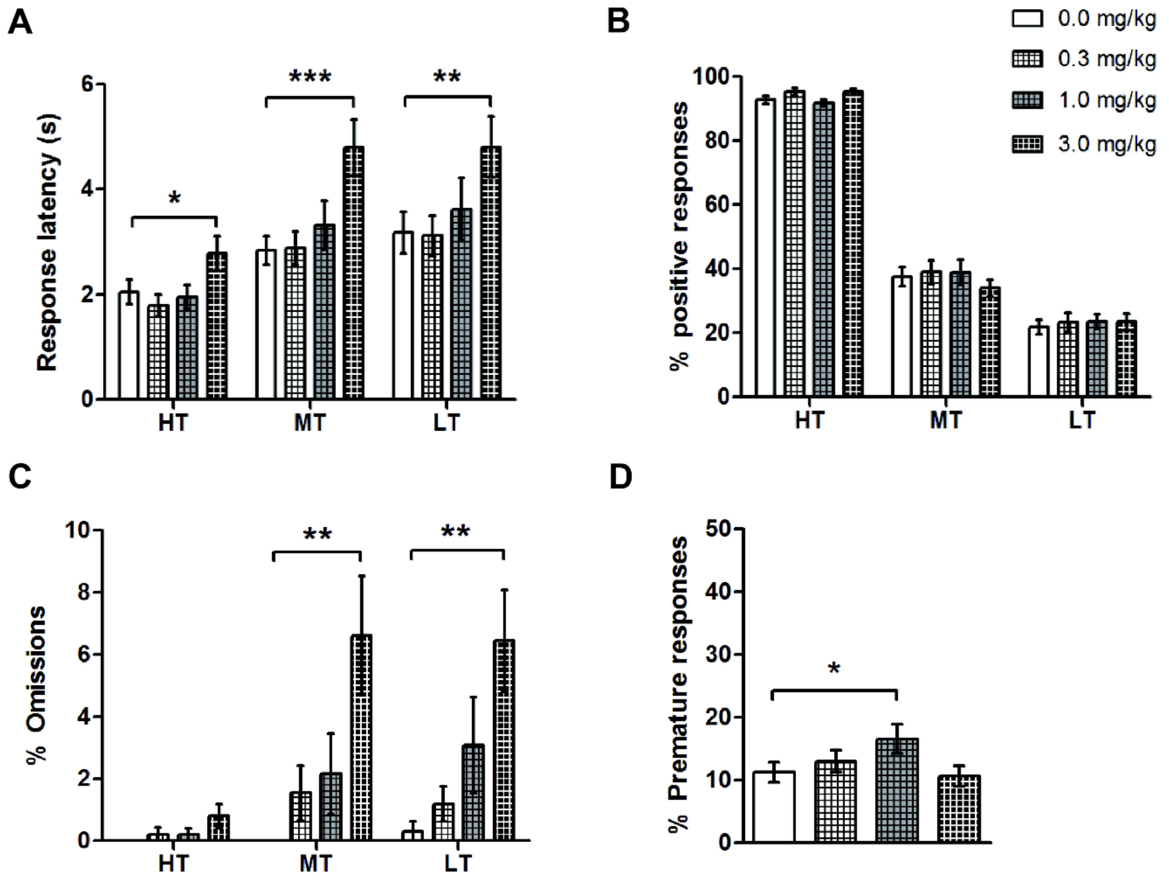

Supplementary Figure 3 – *Behavioural data from the judgement bias task following acute treatment with phencyclidine.*

Acute doses of PCP (0.0, 0.3, 1.0, 3.0 mg/kg) were administered by intraperitoneal injection to measure their effect on judgement bias. (A) PCP caused responses latencies to become slower across all three tones for the highest dose (3.0 mg/kg) only (significant session*tone interaction: $F_{4.49,67.41}=3.079$, $p=0.018$ and significant post-hoc tests: $ps \leq 0.019$). (B) PCP did not alter percentage of positive responses. (C) The highest dose of PCP (3.0 mg/kg) caused rats to omit more responses for the midpoint and low reward tones (significant session*tone interaction: $F_{2.76,41.44}=4.101$; $p=0.014$ and significant post-hoc tests: $ps \leq 0.002$). (D) 1.0 mg/kg PCP caused an increase in percentage of premature responses (main effect of session: $F_{3,45}=2.880$, $p=0.046$ and significant pairwise comparison against vehicle: $p=0.050$). Data represent mean ± SEM. n=16, 40 min pre-treatment. ***$p<0.001$, **$p<0.01$, *$p<0.05$. HT - high reward tone; MT - midpoint tone; LT - low reward tone.
