# Supplementary material for: Behavioural and computational methods reveal differential effects for how delayed and rapid onset antidepressants effect decision making in rats
Source: Eur Neuropsychopharmacol. 2017 Dec;27(12):1268–80. doi: 10.1016/j.euroneuro.2017.09.008 (PMC5720479; doi:10.1016/j.euroneuro.2017.09.008)
Supplement: Supplementary file 9 — Supplementary material [file mmc9.pdf]

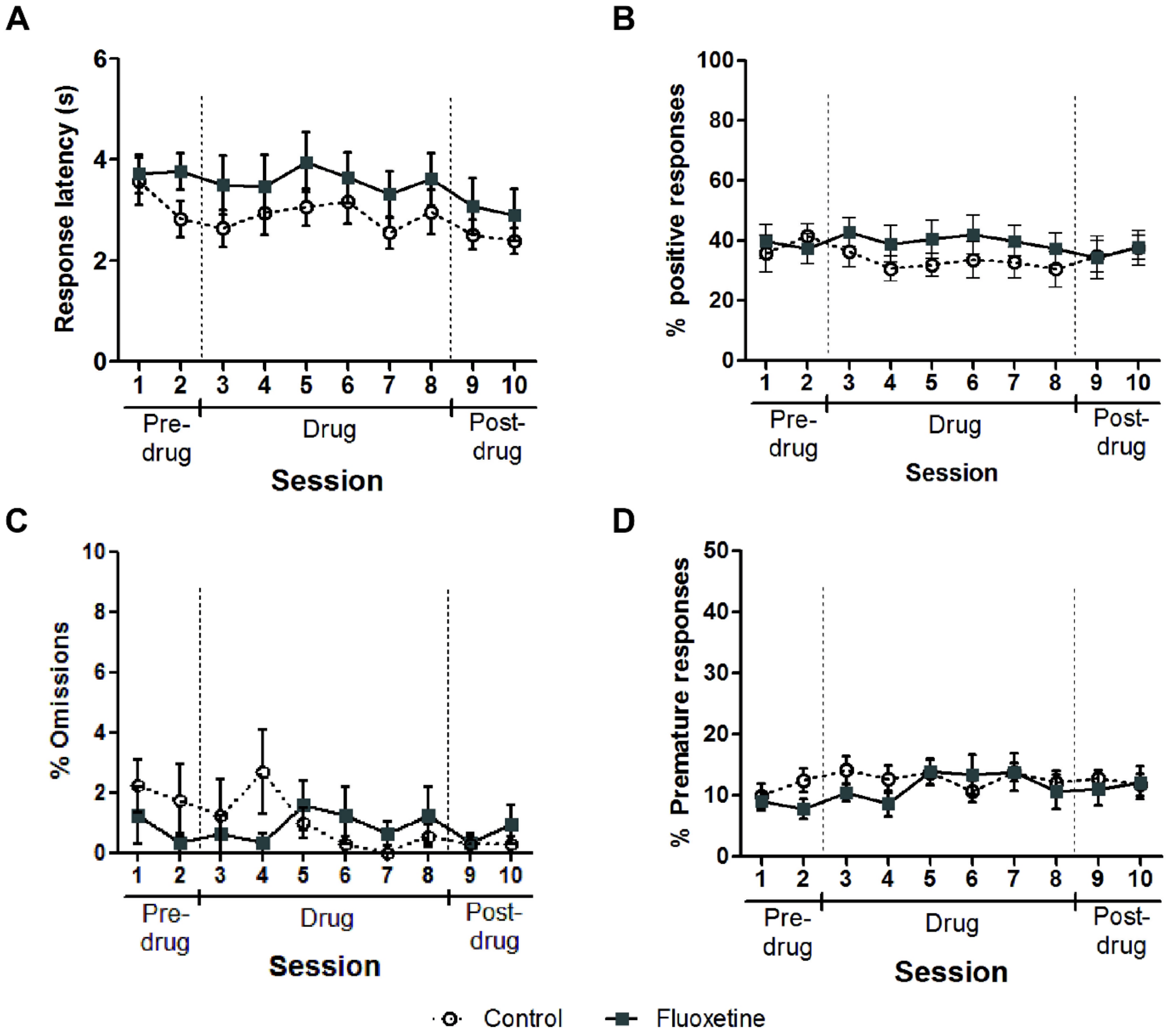

Supplementary Figure 4 – *Behavioural data from the judgement bias task following chronic treatment with an antidepressant.*

Rats in the fluoxetine (control) group were administered daily with fluoxetine (saline vehicle) to measure the effect of chronic treatment with an antidepressant on judgement bias. There were no significant differences between groups during the pre-drug period for any measure. (A/B/C/D) Chronic treatment with fluoxetine had no effect on response latency, percentage positive responses, percentage omissions or percentage of premature responses. Data represent mean ± SEM. Control group: n=9, fluoxetine-treated group: n=8.
