# Supplementary material for: Behavioural and computational methods reveal differential effects for how delayed and rapid onset antidepressants effect decision making in rats
Source: Eur Neuropsychopharmacol. 2017 Dec;27(12):1268–80. doi: 10.1016/j.euroneuro.2017.09.008 (PMC5720479; doi:10.1016/j.euroneuro.2017.09.008)
Supplement: Supplementary file 10 — Supplementary material [file mmc10.pdf]

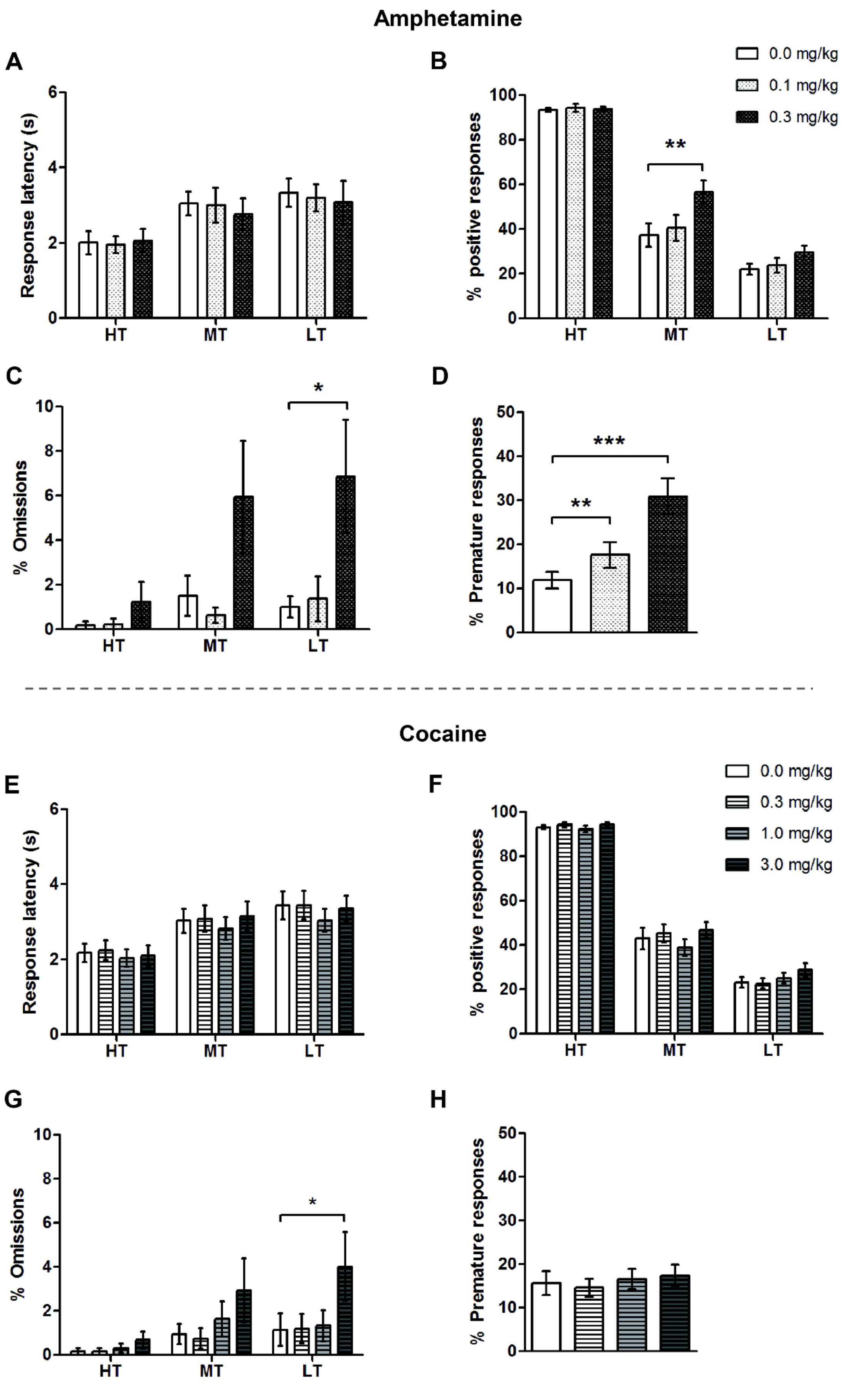
Amphetamine
A
Response latency (s)
6
4
2
0
HT
MT
LT
B
0.0 mg/kg
0.1 mg/kg
0.3 mg/kg
% positive responses
100
80
60
40
20
0
**
HT
MT
LT
C
% Omissions
10
8
6
4
2
0
*
HT
MT
LT
D
% Premature responses
50
40
30
20
10
0
***
**
Cocaine
E
Response latency (s)
6
4
2
0
HT
MT
LT
F
0.0 mg/kg
0.3 mg/kg
1.0 mg/kg
3.0 mg/kg
% positive responses
100
80
60
40
20
0
HT
MT
LT
G
% Omissions
10
8
6
4
2
0
*
HT
MT
LT
H
% Premature responses
50
40
30
20
10
0

Supplementary Figure 5 – *Behavioural data from the judgement bias task following acute treatment with amphetamine or cocaine.*

Acute doses of amphetamine (0.0, 0.1, 0.3 mg/kg) or cocaine (0.0, 0.1, 0.3 mg/kg) were administered by intraperitoneal injection to measure their effect on judgement bias. (A) Amphetamine had no effect on response latency. (B) There was an increase in percentage of positive responses for 0.3 mg/kg amphetamine (significant session*tone interaction: $F_{4,56}=6.033$, $p<0.001$ and significant post-hoc test compared to vehicle: $p=0.001$). (C) The same dose (0.3 mg/kg) caused an increase in omissions for the low reward tone (significant session*tone interaction: $F_{1.85,25.92}=3.713$, $p=0.041$ and significant post-hoc tests: $p=0.037$). (D) Both doses of amphetamine increased percentage of premature responses (main effect of session: $F_{1.46,20.47}=24.515$, $p<0.001$ and significant pairwise comparisons: ps≤0.004). (E/F) Cocaine had no effect on response latency or the percentage of positive responses. (G) The highest dose of cocaine (3.0 mg/kg) caused an increase in omissions for the low reward tone (main effect of session: $F_{2.37,37.83}=3.413$, $p=0.036$ and significant post-hoc tests: $p=0.035$). (H) Cocaine did not alter the percentage of premature responses. Data represent mean ± SEM. Amphetamine: n=15, 15 min pre-treatment. Cocaine: n = 17, 10 min pre-treatment. ***p<0.001, **p<0.01, *p<0.05. HT - high reward tone; MT - midpoint tone; LT - low reward tone.
