# Supplementary material for: Behavioural and computational methods reveal differential effects for how delayed and rapid onset antidepressants effect decision making in rats
Source: Eur Neuropsychopharmacol. 2017 Dec;27(12):1268–80. doi: 10.1016/j.euroneuro.2017.09.008 (PMC5720479; doi:10.1016/j.euroneuro.2017.09.008)
Supplement: Supplementary file 11 — Supplementary material [file mmc11.pdf]

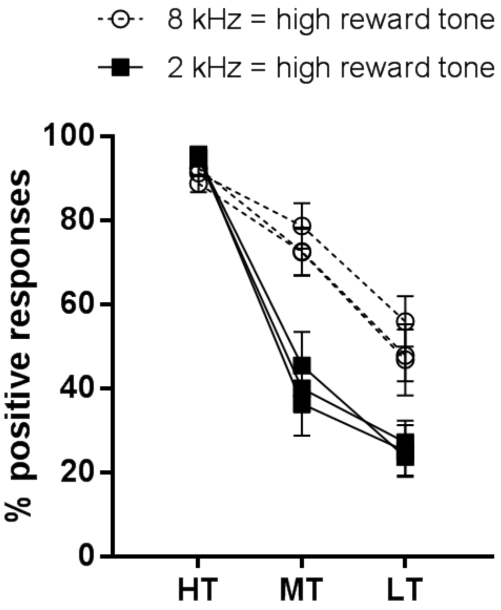

Supplementary Figure 6 – *Baseline probe session data from cohort 1.*
The percentage of positive responses made for each tone during probe test sessions where no experimental manipulation occurred, split to show responding of rats that had the 8 kHz tone paired with the high value reward, compared to rats who had the 2 kHz tone paired with high value reward. Although responding is the same for the high reward tone in both groups, it is clear that responding on the midpoint and low reward tone is biased towards more positive responding in the 8 kHz = high reward rats, suggesting an innate preference for this tone. This also demonstrates why a large number of animals had to be excluded from analysis in cohort I, as most rats who had 8 kHz as the high reward tone failed to maintain > 60% accuracy in responding for the low reward tone. HT – high tone; MT – midpoint tone; LT – low tone. n = 8 per group.
